# Early prediction of mortality upon intensive care unit admission

**DOI:** 10.1186/s12911-024-02807-6

**Published:** 2024-12-18

**Authors:** Yu-Chang Yeh, Yu-Ting Kuo, Kuang-Cheng Kuo, Yi-Wei Cheng, Ding-Shan Liu, Feipei Lai, Lu-Cheng Kuo, Tai-Ju Lee, Wing-Sum Chan, Ching-Tang Chiu, Ming-Tao Tsai, Anne Chao, Nai-Kuan Chou, Chong-Jen Yu, Shih-Chi Ku

**Affiliations:** 1 Department of Anesthesiology, National Taiwan University Hospital, No 7, Chung Shan South Road, Taipei, Taiwan; 2 Taiwan AI Labs, Taipei, Taiwan; 3 Department of Computer Science and Information Engineering, National Taiwan University, No.1, Sec. 4, Roosevelt Road, Taipei, Taiwan; 4 Graduate Institute of Biomedical Electronics and Bioinformatics, National Taiwan University, No.1, Sec. 4, Roosevelt Road, Taipei, Taiwan; 5 Department of Internal Medicine, National Taiwan University Hospital, No 7, Chung Shan S. Road, Taipei, Taiwan; 6 Department of Anesthesiology, Far Eastern Memorial Hospital, No. 21, Sec. 2, Nanya S. Rd, New Taipei, Taiwan; 7 Department of Surgery, National Taiwan University Hospital, No.7, Chung Shan S. Rd, Taipei, Taiwan; 8 Department of Internal Medicine, National Taiwan University Hospital, Hsin-Chu Branch, No. 25, Ln. 442, Sec. 1, Jing-Guo Rd., North Dist, Hsinchu City, Taiwan

**Keywords:** Intensive care, Critically ill, Mortality, Prediction

## Abstract

**Background:**

We aimed to develop and validate models for predicting intensive care unit (ICU) mortality of critically ill adult patients as early as upon ICU admission.

**Methods:**

Combined data of 79,657 admissions from two teaching hospitals’ ICU databases were used to train and validate the machine learning models to predict ICU mortality upon ICU admission and at 24 h after ICU admission by using logistic regression, gradient boosted trees (GBT), and deep learning algorithms.

**Results:**

In the testing dataset for the admission models, the ICU mortality rate was 7%, and 38.4% of patients were discharged alive or dead within 1 day of ICU admission. The area under the receiver operating characteristic curve (0.856, 95% CI 0.845–0.867) and area under the precision-recall curve (0.331, 95% CI 0.323–0.339) were the highest for the admission GBT model. The ICU mortality rate was 17.4% in the 24-hour testing dataset, and the performance was the highest for the 24-hour GBT model.

**Conclusion:**

The ADM models can provide crucial information on ICU mortality as early as upon ICU admission. 24 H models can be used to improve the prediction of ICU mortality for patients discharged more than 1 day after ICU admission.

**Supplementary Information:**

The online version contains supplementary material available at 10.1186/s12911-024-02807-6.

## Background

Predicting the outcomes of critically ill patients may provide valuable information for decision-making and resource allocation in intensive care unit (ICU) settings [1–4]. Developing a predictive model must use variables obtained before the prediction time of the model [5–7]. The prediction time of most mortality prediction models for critically ill patients is at 24 h after ICU admission [8–18]. We notice that a considerable proportion of patients were discharged alive or dead within 24 h of ICU admission, and mortality of these patients could not be predicted by a model developed to make a prediction at 24 h or later after ICU admission. To date, only a few machine learning models were developed to early predict mortality of ICU patients at 1 to 6 h after ICU admission [19–22]. Therefore, we developed machine learning models to predict ICU mortality of critically ill patients upon ICU admission and investigated the model performance. Moreover, these models were interpreted by feature importance and personalized risk assessment. For patients discharged more than 1 day after ICU admission, we developed machine learning models to predict ICU mortality of critically ill patients at 24 h after ICU admission and compared the model performance with conventional severity score models.

## Materials and methods

### Study design and data source

We developed and validated the admission models (ADM models) and 24-hour models (24 H models) to predict ICU mortality upon ICU admission and at 24 h after ICU admission, respectively, by using data of adult patients from two ICU databases. The first database was the Medical Information Mart for Intensive Care (MIMIC)-IV, an open-access, anonymized database of 76,540 admissions from 2008 to 2019 at a Boston teaching hospital [23, 24]. Three members in our team have finished the Collaborative Institutional Training Initiative training and examination (Certification Number: 32697132 for YCY, 43101861 for KCK, 39956855 for YWC) and have been approved to access the MIMIC-IV database. The second database was the Center of Outcome and Resource Evaluation (CORE), an anonymized database of 11,966 admissions between 2019 and 2020 at a Taipei teaching hospital, which is a branch of multi-center databases of Taiwan CORE. Taiwan CORE is collaborating with the Australian and New Zealand Intensive Care Society CORE adult patient database [25, 26]. Using CORE datasets was approved by the hospital Research Ethics Committee (REC number 202004016RINB) and registered on the ClinicalTrials.gov protocol registration system (ID NCT04541849) on 09/09/2020. Written informed consent was waived because of the retrospective nature and deidentification of protected health information in accordance with the Health Insurance Portability and Accountability Act Privacy Rule [27]. This study was conducted and reported according to the Transparent Reporting of a Multivariable Prediction Model for Individual Prognosis or Diagnosis (TRIPOD) statement [28] and the recommended guidance [7]. Length of stay (LOS) in ICU was calculated as ICU discharge date minus ICU admission date.

### Population and datasets

For patients with multiple ICU admissions, we only included their first ICU admission in the same hospitalization. The exclusion criteria included an age of < 18 or < 20 years upon ICU admission for patients in the MIMIC − IV and CORE databases, respectively, and patients with a death date error in the MIMIC − IV database. The TRIPOD statement recommends that a nonrandom split of data by time is more effective for evaluating model performance because it allows for the influence of nonrandom variation between the data sets [19]. Therefore, to develop the ADM models, we allocated the records of patients admitted between 2008 and 2016 from the MIMIC − IV database and between 2019 and July 2020 from the CORE database into the ADM training data set. To validate the ADM models, we allocated the records of patients admitted between 2017 and 2019 from the MIMIC − IV database and between August 2020 and December 2020 from the CORE database into the ADM testing dataset. When developing and validating the 24 H models, we excluded patients discharged within 1 day (on day 0 or 1) of ICU admission and allocated the remaining records into the 24 H training and testing data sets.

### Data preprocessing

We extracted the data from the MIMIC − IV database by using pgAdmin4 (version 5.2, the pgAdmin Development Team) with PostgreSQL 10. We utilized the codes available on the GitHub repository (https://github.com/MIT-LCP/mimic-code/tree/main/mimic-iv) for data extraction and processing [29]. We perform additional data processing by using the graphical user interface platform of RapidMiner Studio (Version 9.10, RapidMiner, Boston, United States) [30–32]. We extracted the following variables from both databases: demographic characteristics (e.g., age, weight, and body mass index), premorbid status (e.g., metastasis, end-stage renal disease, coronary arterial disease, dementia, chronic heart failure), treatments, Glasgow coma scale (GCS), vital signs measurements, and laboratory data from the first 24 h after ICU admission. When training the ADM models, only the variables obtained before or upon ICU admission could be used as features. When training the 24 H models, only the variables obtained before or at 24 h after ICU admission could be used as features. We checked all the data for outliers and errors by using a frequency histogram, and errors were corrected when possible (e.g., conversion of units of laboratory data). Missing vital signs measurements and GCS were imputed with neighboring values from the nearest time points. Missing demographics information and laboratory data were imputed using the mean or median values from their original databases as appropriate.

### Outcomes

The outcome to be predicted was ICU mortality, which was defined as a death occurring during the ICU stay or an urgent discharge home of imminently dying patients expressing a wish to die at home.

### Feature selection and modeling

We developed and validated the models by using the RapidMiner Studio platform (Version 9.10, RapidMiner). We trained the models by using three machine learning algorithms: logistic regression (LR), gradient boosted trees (GBT), and deep learning (DL). We perform 10-fold cross-validation method for model training with the ADM and 24 H training data sets. The optimized parameter operator was used to find out the optimal parameter set and determine the best model for GBT models training. When training the ADM models, we extracted a set of 18 features, including demographic characteristics, premorbid status, first GCS score and vital signs measurements obtained upon ICU admission on the basis of their clinical relevance and our analysis of the baseline descriptive statistics [33]. When training the 24 H models, we extracted a set of 40 features, including demographic characteristics, premorbid status, GCS scores, vital signs measurements, diagnoses, treatments, and laboratory data obtained within the first 24 h after ICU admission on the basis of their clinical relevance and our analysis of the baseline descriptive statistics [33]. In addition, LR algorithm was used to predict ICU mortality with conventional severity score system, sequential organ failure assessment (SOFA) score and acute physiology score (APS) of acute physiologic assessment and chronic health evaluation (APACHE) III [34, 35], respectively, at 24 h after ICU admission. The ADM and 24 H testing data sets were used for model validation. We interpreted the models based on the ranking and weight of feature importance, which were calculated using the Explain Prediction operator in RapidMiner Studio platform (Version 9.10, RapidMiner). This operator derives the weights directly from the model explanations. When true labels are available for the test data, weights are adjusted based on the local explanations: supporting explanations for correct predictions increase the weights, whereas contradicting explanations for incorrect predictions also contribute positively to the weights. We generated a personalized risk assessment and model simulator by using the RapidMiner Studio platform (Version 9.10, RapidMiner).

### Statistical analysis

Regarding statistical analysis, the categorical variables are expressed as *n* (%) and were compared using a χ^2^ test. The continuous variables are expressed as medians (interquartile ranges [IQRs]) and were compared using the Mann–Whitney *U* Test. A two-sided *p* value of < 0.05 was considered statistically significant. Adjustments for multiplicity were not performed due to the large sample size and the presence of highly significant results (most *p*-values < 0.001), which may diminish the risk of false-positive findings. Results with *p*-values ranging from 0.01 to 0.05 should be interpreted with caution. We performed all the statistical analysis by using statistical software (SPSS 27; IBM SPSS, USA).

We assessed the performance of the models in terms of both discrimination and calibration by using their AUROC values, area under the precision-recall curve (AUPRC) values, sensitivity, precision, specificity, Brier score, and calibration plots. Performance measures were reported with their 95% confidence intervals (CIs). AUPRC is more informative than the AUROC when evaluating binary classifiers on imbalanced data [36]. We compared the AUROC and AUPRC values by using Python (version 3.7.3, Python Software Foundation, Delaware, United States) and the scikit-learn package (version 0.23.1). The calibration plots were drawn using the R software (version 4.1.3, Foundation for Statistical Computing, Vienna, Austria). To investigate the progressive decay of the AUROC values of each model, we calculated the daily AUROC values for those patients who remained in the ICU on day 0 to 14 of ICU admission.

## Results

### Data set information

**Fig. 1:**
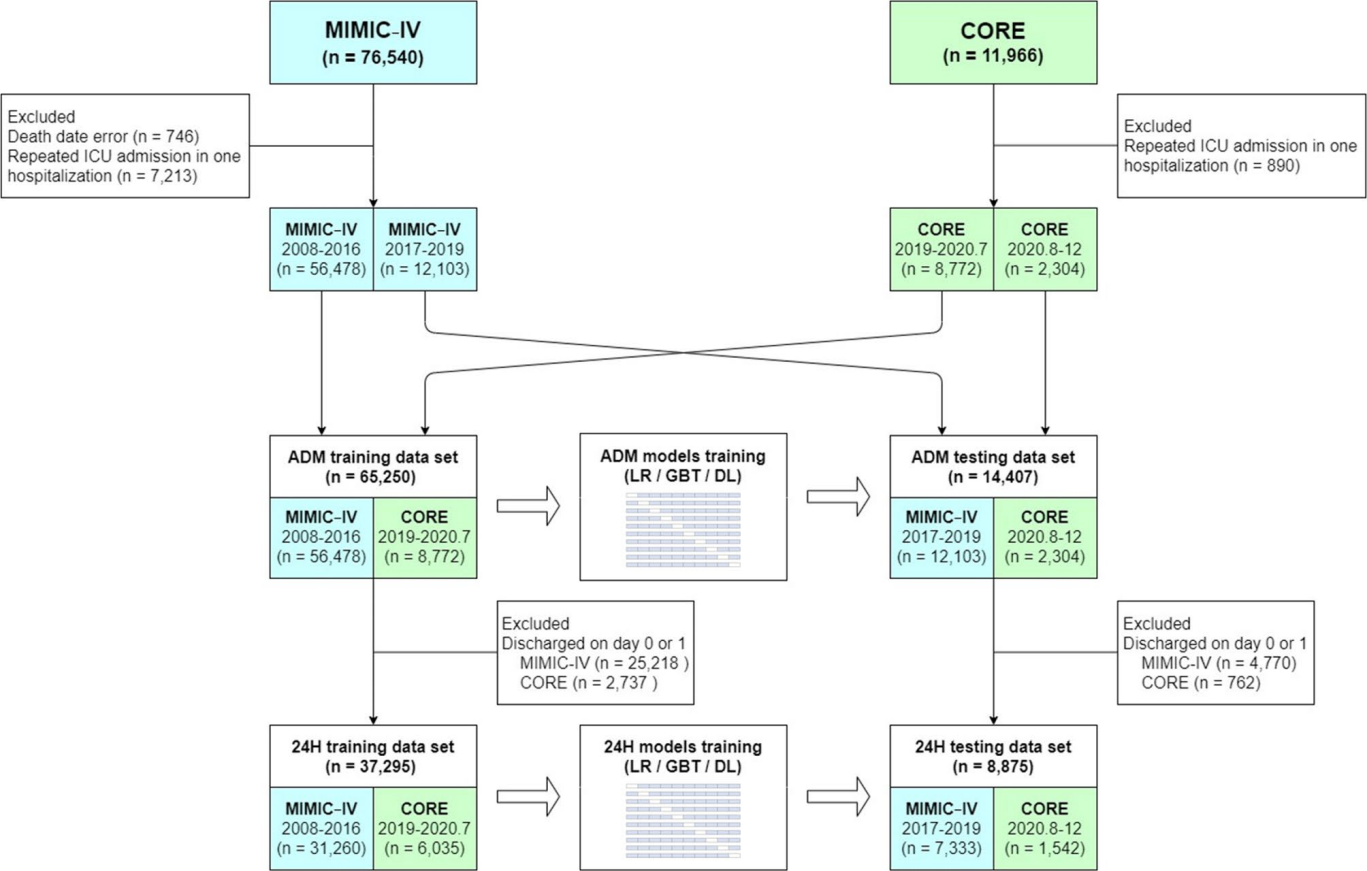
Flowchart of patient inclusion criteria and allocation to training and testing data sets. *ADM* admission, *CORE* Center of Outcome and Resource Evaluation critical care database, *DL* deep learning, *GBT* gradient boosting trees, *ICU* intensive care unit, *LR* logistic regression, *MIMIC* Medical Information Mart for Intensive Care database

We used 65,250 and 14,407 admission records to develop and validate the performance of the ADM models, respectively (Fig. 1). The clinical characteristics of the survivors and nonsurvivors in the ADM training and testing data sets are presented in Additional file 1: Table S1; the ICU mortality rate in the training and testing data sets were 6.6% and 7%, respectively. Of the 14,407 admissions in the ADM testing data set, 38.4%, 49.5%, and 12.1% were discharged alive or dead within 1 day, within 2 to 7 days, and more than 7 days after ICU admission, respectively. The clinical characteristics of patients from the MIMIC − IV and CORE databases in the ADM testing data set are presented in Table 1. The number of patients with missing data from the MIMIC − IV and CORE databases in the ADM testing data set are presented in Additional file 1: Table S2. We used 37,295 and 8,875 admission records to develop and validate the performance of the 24 H models, respectively (Fig. 1). The clinical characteristics of the survivors and nonsurvivors in the 24 H training and testing data sets are presented in Additional file 1: Table S3. The clinical characteristics of the patients from the MIMIC − IV and CORE databases in the 24 H testing data set are presented in Additional file 1: Table S4. The number of patients with missing data from the MIMIC − IV and CORE databases in the 24 H testing data set are presented in Additional file 1: Table S5.

**Table 1:** Characteristics of patients from the MIMIC − IV and CORE data in the ADM data sets

	Training		Testing	
	MIMIC − IV	CORE	MIMIC − IV	CORE
Number	56,478	8,772	12,103	2,304
Age (years)	66 (54–78)	67 (56–77)	66 (54–76)	67 (56–77)
Female (%)	25,289 (44.8%)	3,527 (40.2%)	5,088 (42%)	883 (38.3%)
Height (cm)	170 (163–178)	162 (156–168)	170 (163–178)	162 (156–168)
Weight (kg)	77.8 (65.1–92.6)	62.4 (53.8–70.1)	78.5 (66-93.3)	62.5 (54.1–70)
CAD	9,133 (16.2%)	1,260 (14.4%)	2,160 (17.8%)	364 (15.8%)
CHF	16,121 (28.5%)	486 (5.5%)	2,897 (23.9%)	129 (5.6%)
ESRD	12,590 (22.3%)	587 (6.7%)	2,026 (16.7%)	138 (6%)
Diabetes	17,200 (30.5%)	26,24 (29.9%)	3,230 (26.7%)	690 (29.9%)
Dementia	1,866 (3.3%)	256 (2.9%)	666 (5.5%)	65 (2.8%)
Metastatic cancer	3,511 (6.2%)	873 (10%)	736 (6.1%)	101 (4.4%)
Postoperative care	7,496 (13.3%)	4,374 (49.9%)	2,051 (16.9%)	1,129 (49%)
Elective operation	3,509 (6.2%)	3,263 (37.2%)	954 (7.9%)	899 (39%)
Pre-ICU hospital stay	0 (0–0)	2 (1–4)	0 (0–1)	2 (1–4)
**Upon ICU admission**				
Glasgow Coma Scale	15 (10–15)	15 (13–15)	15 (7–15)	15 (14–15)
Body temperature (℃)	36.7 (36.4–37.1)	36.3 (35.8–36.8)	36.8 (36.5–37.1)	36.3 (35.7–36.7)
Respiratory rate (bpm)	18 (15–22)	19 (15–22)	18 (16–22)	19 (15–22)
Heart rate (bpm)	86 (75–101)	91 (78–107)	84 (74–99)	90 (77–105)
SBP (mm Hg)	124 (108–142)	134 (116–155)	123 (108–140)	136 (117–155)
DBP (mm Hg)	678 (57–79)	71 (61–83)	69 (59–82)	71 (61–83)
MAP (mm Hg)	87 (76–99)	93 (81–106)	88 (77–100)	94 (82–107)
SOFA score	4 (2–6)	6 (4–10)	4 (2–7)	7 (5–10)
APS	41 (31–56)	55 (41–77)	37 (27–53)	56 (42–76)
ICU mortality	3,572 (6.3%)	753 (8.6%)	803 (6.6%)	209 (9.1%)
Hospital Mortality	5,542 (9.8%)	1,404 (16%)	1,203 (9.9%)	344 (14.9%)
LOS in ICU (days)	2 (1–3)	3 (1–6)	2 (1–4)	2 (1–5)
LOS in hospital (days)	6 (4–11)	16 (8–32)	7 (4–12)	15 (7–28)

Values are presented as numbers (%) or medians (interquartile ranges). *APS* acute physiology score of the acute physiologic assessment and chronic health evaluation (APACHE) III, *bpm* beats or breaths per minute for heart rate and respiratory rate, respectively, *CAD* coronary artery diseases, *CHF* congestive heart failure, *CORE* Center of Outcome and Resource Evaluation critical care database, *DBP* diastolic blood pressure, *ESRD* end-stage renal disease, *ICU* intensive care unit, *LOS* length of stay, *MAP* mean arterial pressure, *MIMIC* Medical Information Mart for Intensive Care, *SBP* systolic blood pressure, *SOFA* sequential organ failure assessment

### Performance and feature importance of the ADM and 24 H models

**Fig. 2:**
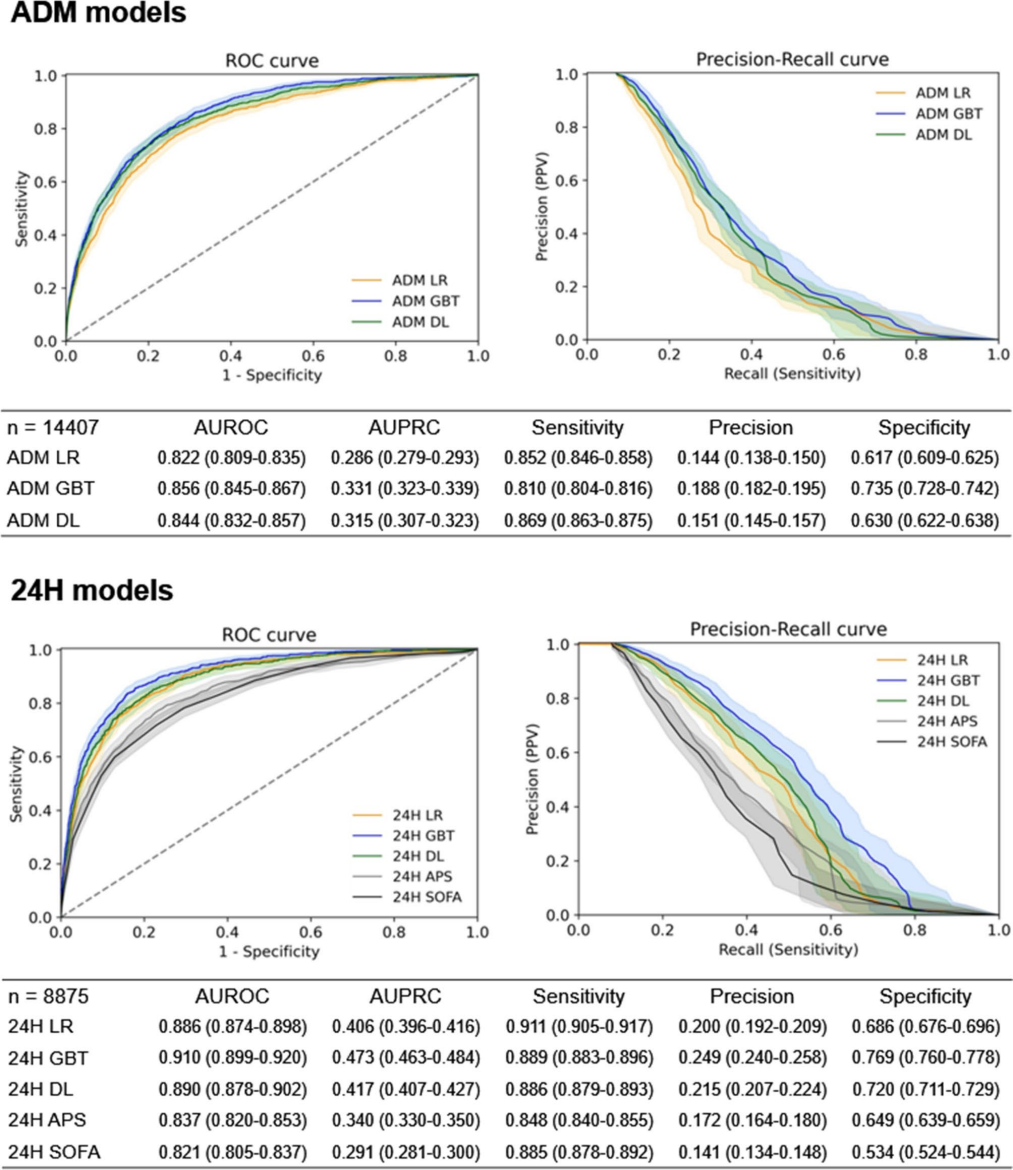
Performance of the ADM and 24 H models in the testing data sets. Performance measures are presented as values with corresponding 95% confidence intervals. The cutoff value for sensitivity, precision, and specificity was 0.04 for all models. *ADM* admission, *APS* acute physiology score of the acute physiologic assessment and chronic health evaluation (APACHE) III, *AUPRC* area under the precision-recall curve, *AUROC* area under the receiver operating characteristic curve, *DL* deep learning, *GBT* gradient boosting trees, *LR* logistic regression, *SOFA* sequential organ failure assessment

**Fig. 3:**
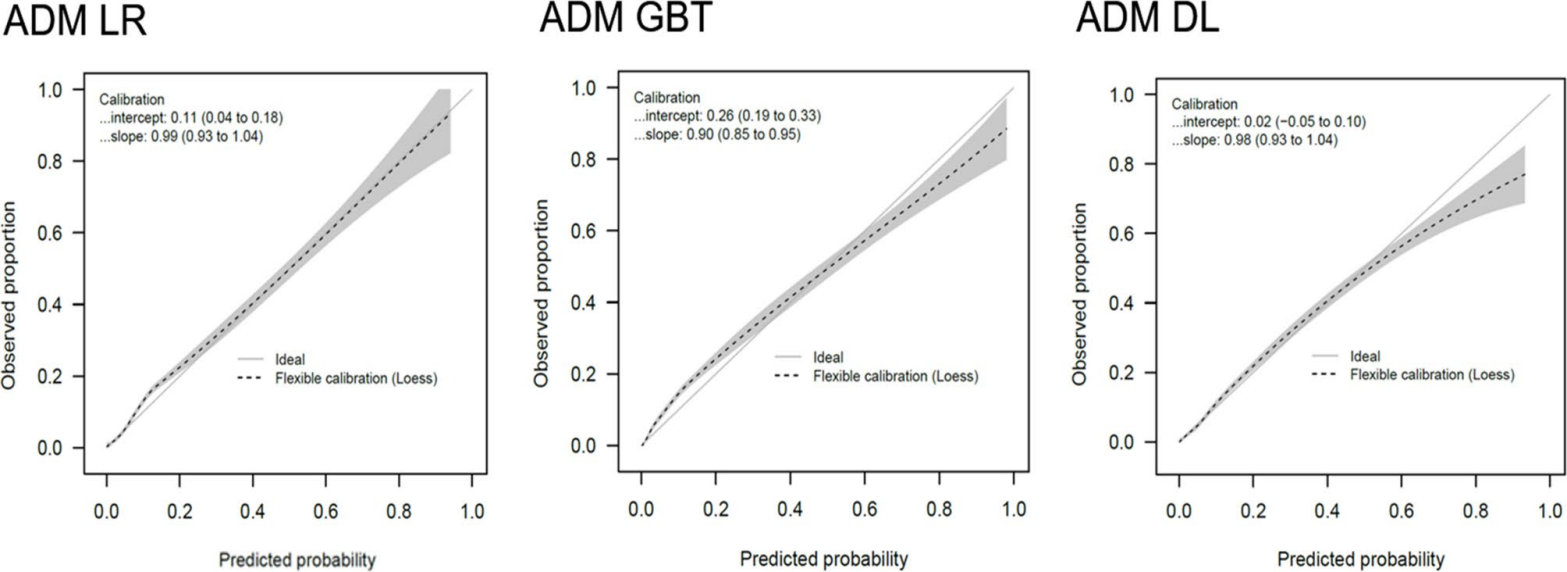
Calibration plots of the ADM models. *ADM* admission, *DL* deep learning, *GBT* gradient boosting trees, *LR* logistic regression

**Fig. 4:**
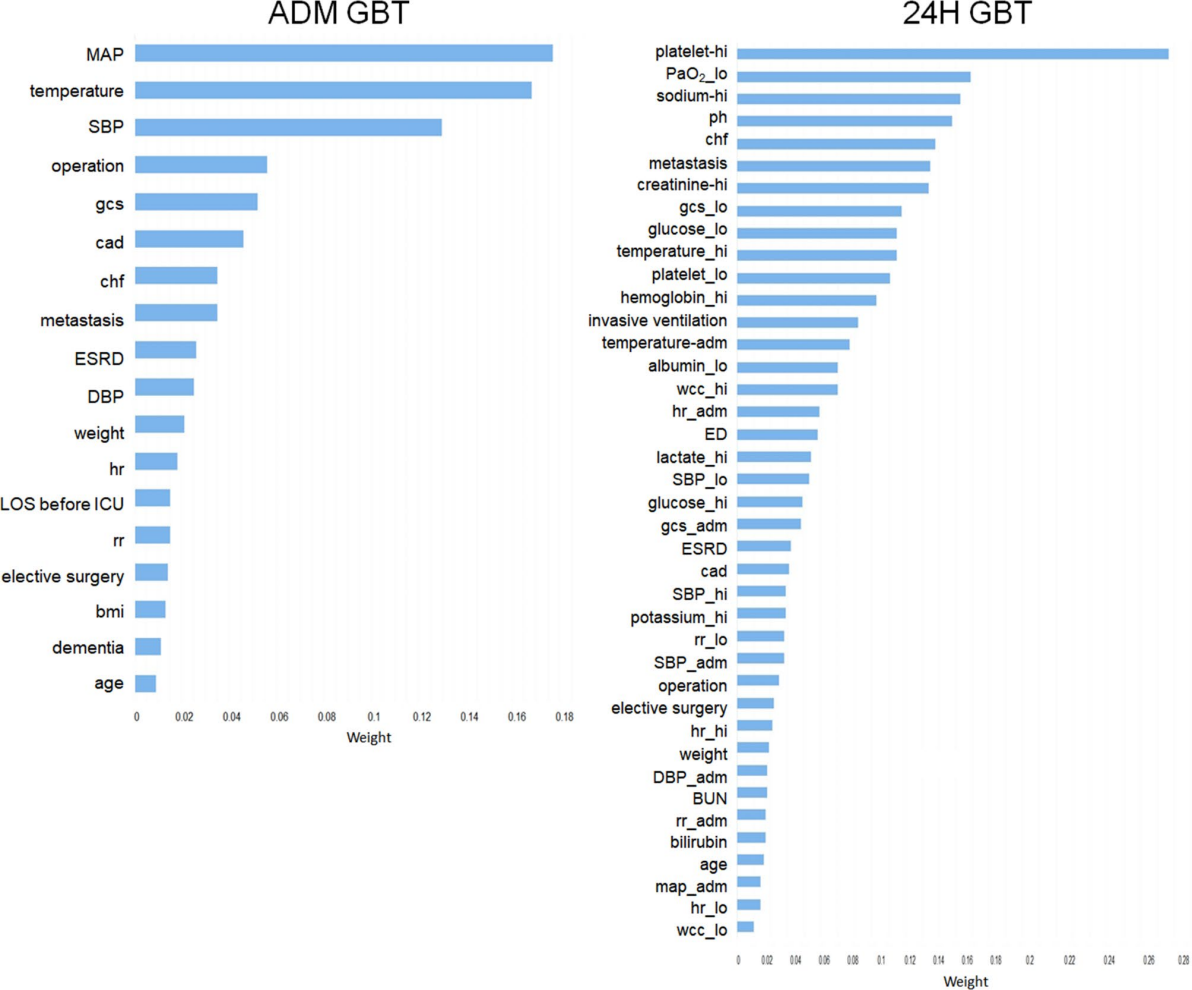
Feature importance of gradient boosting trees models. *ADM* admission, *bmi* body mass index, *BUN* blood urine nitrogen, *cad* coronary artery diseases, *chf* congestive heart failure, *DBP* diastolic blood pressure, *ED* emergency department, *ESRD* end stage renal disease, *GBT* gradient boosting trees, *gcs* Glasgow coma scale, *hr* heart rate, *ICU* intensive care unit, *inv* invasive ventilation, *LOS* length of stay, *MAP* mean arterial pressure, *ph* power of hydrogen, *plat* platelet, *rr* respiratory rate, *SBP* systolic blood pressure, *wcc* white cell counts

The model performance indices during training are presented in the Additional file 1: Figure S1. The model performance indices of the ADM and 24 H models in the testing data set are presented in Fig. 2. Regarding the ADM models, the AUROC (0.856, 95% CI 0.845–0.867) and AUPRC (0.331, 95% CI 0.323–0.339) of the ADM GBT model were higher than those of the ADM LR and DL models. Regarding the 24 H models, the AUROC (0.910, 95% CI 0.899–0.920) and AUPRC (0.473, 95% CI 0.463–0.484) of the 24 H GBT model were again higher than those of the 24 H LR, DL, APS, and SOFA models. The calibration plots of the ADM models are presented in Fig. 3. The intercepts and slopes of the calibration plots were 0.26 and 0.9, respectively, for the ADM GBT. The Brier scores of the ADM LR, GBT, and DL models were 0.087, 0.085, and 0.084, respectively. The calibration plots of the 24 H models are presented in Additional file 1: Figures S2. The ranking and weights of the features of the ADM and 24 H models with GBT algorithm are presented in Fig. 4. The ranking and weights of the features of the ADM and 24 H models with LR and DL algorithms are presented in Additional file 1: Figures S3 and S4. For the ADM models, mean arterial pressure, body temperature, postoperative intensive care, and GCS were important features. For the 24 H models, platelet count, white cell count, invasive ventilation, body temperature, and GCS were important features. The AUROC values of the ADM and 24 H models and mortality rate of those patients who remained in the ICU on day 0 to 14 of ICU admission are presented in Additional file 1: Figures S5. The AUROC values of the ADM and 24 H models decayed over time.

The performance of models trained with different ADM and 24 H training datasets on various ADM and 24 H testing datasets is detailed in Additional file 1: Tables S6 and S7, respectively. For both ADM and 24 H models, models trained with hybrid training datasets exhibited superior performance on hybrid testing datasets. In the MIMIC ADM testing dataset, the AUROC of GBT models was higher for model trained with the hybrid dataset compared to that trained with the MIMIC training dataset. Similarly, in the MIMIC 24 H testing dataset, the AUROC for both GBT and DL models was higher for models trained with the hybrid dataset than for those trained with the MIMIC training dataset.

### Personalized risk assessment in a model simulator

An example of personalized risk assessment was demonstrated with a model simulator using the ADM GBT model in Additional file 1: Figure S6. In the example, the predicted risk of ICU mortality was 21%. The identified risk factors were mild tachycardia, tachypnea, an emergent operation, and a long hospital stay before ICU admission. If the value of any risk factor was changed, the simulator recalculated the predicted risk accordingly.

## Discussion

A total of 38.4% of the admissions in the ADM testing data set were discharged alive (36.3%) or dead (2.1%) within 1 day; therefore, the 24 H and conventional models could not be used to predict ICU mortality in these patients. The ADM models could predict an individual’s risk of ICU mortality on the basis of their demographic characteristics, premorbid status, GCS score, and vital signs measurements immediately after ICU admission with good discrimination and calibration. Compared to ADM machine learning models and conventional APS and SOFA models, subsequent 24 H machine learning models can improve the predictability of ICU mortality among patients discharged alive or dead more than 1 day after ICU admission.

Our findings suggest that training machine learning models to predict ICU mortality upon ICU admission is feasible with good predictability. This enables critical care teams to identify patients at risk of acute death within 1 day after ICU admission as early as possible. Additionally, the training times and predictive accuracy clearly favor the use of ML models over traditional scores. LR models train in seconds, and GBT models take minutes, but GBT’s superior performance justifies the increased complexity. Our ADM model provides critical immediate insights upon ICU admission, an advantage over scores like APS and SOFA, which are calculated after 24 h. Furthermore, GBT, DL, and LR outperform APS and SOFA in the 24-hour models, demonstrating greater predictive power and operational efficiency without the need for manual data abstraction, unlike APACHE. These advantages make our models highly suitable for fast-paced clinical settings. Moreover, we examined the AUROC values of the ADM on different days after ICU admission. The AUROC values of all the ADM models decreased over time, reaching < 0.8 on day 3 and < 0.7 on day 6 after ICU admission. Numerous factors can affect a model to predict a patient’s risk of ICU mortality, including treatment decision, treatment response, the severity and reversibility of organ injuries, complications, resuscitation resources, and socioeconomic status [37–39]. Furthermore, our results revealed that using the 24 H models instead of the ADM models to predict ICU mortality among patients who have been in the ICU for over 1 day can improve the predictability.

In this study, we assessed the feature importance of model. We suggest that visualizing feature importance can help critical care teams understand how machine learning models make decisions [40, 41], and help them determine whether a given model is suitable for predicting ICU mortality among their patients. Regarding calibration plots, if a model has poor calibration, it must be recalibrated to ensure accurate prediction [42]. The 24 H prediction model incorporates features also utilized in the APACHE score, including temperature, MAP, heart rate, sodium, potassium, creatinine, PaO_2_, pH, glucose, WCC, GCS, age, and chronic conditions like CHF and ESRD, as well as features from the SOFA score such as platelet count and MAP. These features focus on key physiological and organ function indicators, ensuring a comprehensive assessment of patient’s condition. By aligning with established scoring systems like APACHE and SOFA, the model leverages proven metrics to enhance the accuracy and reliability of its predictions. In the ADM GBT model, CHF and CAD have moderate weights, with Metastasis, ESRD, and Dementia contributing at lower weights compared to primary features like MAP, temperature, and SBP. In the 24 H GBT model, CHF and Metastasis become more important, but chronic health conditions overall still have moderate weights relative to acute physiological variables.

The relationship between ICU mortality and feature severity presents two important considerations. When patients show less severe clinical features, this could indicate either early ICU admission or pre-admission correction of values, both of which influence mortality outcomes. Early ICU admission, reflected in milder clinical presentations, typically leads to better prognosis and reduced mortality. Similarly, correctable feature values upon ICU admission may signify reversible injuries, correlating with improved survival rates. Although pre-ICU data was not included in our analysis, these factors help mitigate lead-time bias in ICU mortality predictions. However, it’s important to note that when predicting longer-term outcomes such as 90-day survival, overall survival time, or comparing standardized mortality ratios, experts caution that lead-time bias may become more significant and should be carefully considered [43–45]. Furthermore, s simulator with visualized personalized risk of ICU mortality may be used to help patients and their families understand the influence of risk factors. Additional studies are warranted to investigate the impact of the personalized risk factors analysis on clinical practice in critical care and the shared decision-making process with patient and their families.

This study has several limitations. First, the vital signs measurements and laboratory data prior to ICU admission were not available in the CORE database, and the information of frailty was not available in the MIMIC − IV database. These variables may improve the performance of the ADM models. Second, the information of medications, fluid supplement, and unstructured data were not available in the CORE database. These variables may improve the performance of the 24 H models. Third, we did not classify the patients into subgroups on the basis of diagnosis. Forth, Fourth, we did not collect data regarding patients’ palliative care status. Palliative care decisions may affect the predictability of the 24 h model, as some patients may choose a palliative care plan. The decision to include this information or exclude these patients should be considered based on the prediction model’s purpose. The ADM model, however, was less affected by palliative care considerations, as the worst values typically indicate a refractory condition that leads to palliative status. Furthermore, we acknowledge that the models proposed herein will likely be replaced by dynamic patient-level prediction models when the electronic medical system can real-time provide massive multivariate time-series data to the deployed models [20, 46]. One key consideration when evaluating prediction models that utilize GCS scores is the management of sedated or ventilated patients. In the MIMIC database, patients who cannot be assessed due to sedation or ventilation but are otherwise neurologically intact are typically assigned a GCS score of 15 [29]. Our database follows similar standards. Additionally, for intubated coma patients, verbal scores are adjusted according to the protocol of our Division of Neurosurgery. Specifically, patients with GCS scores ranging from 2T to 8T receive an additional verbal score of 1. Those with a score of 9T are assigned a total score of 12 if the motor score is 5 or 14 if the motor score is 6. Finally, patients with a score of 10T are assigned a total score of 15. We noted that a practical rule was published after we built the CORE database [47]. However, these rules are not uniformly implemented in most hospital electronic health records. Consequently, sedated patients may receive highly variable GCS scores, ranging from as low as 3 to as high as 15, depending on the care provider. Addressing this inconsistency is crucial for the effective training and deployment of the machine learning model, as it ensures the model’s accuracy, reliability, and ability to generalize across diverse clinical settings.

## Conclusion

More than one-third of the ICU patients in this study were discharged alive or dead within 1 day of ICU admission. Early ADM prediction models can provide crucial information regarding the risk of ICU mortality among such patients upon ICU admission. Subsequent 24 H models can be used to improve predictability of ICU mortality among patients discharged more than 1 day after ICU admission.

## Electronic supplementary material

Below is the link to the electronic supplementary material.

Supplementary Material 1

## Data Availability

MIMIC-IV database is openly available for credentialed users who finished the required training and signed the data use agreement. The CORE data set used during the current study are available from the corresponding author on reasonable request after obtaining the agreement of Research Ethic Committee of National Taiwan University Hospital. All codes and processes used in the current study are available from the corresponding author on reasonable request.

## Abbreviations

24 H: 24 h
ADM: Admission
APACHE: Acute physiologic assessment and chronic health evaluation
APS: Acute physiology score
AUPRC: Area under the precision-recall curve
AUROC: Area under the receiver operating characteristic curve
CI: Confidence interval
CORE: Center of outcome and resource evaluation
DL: Deep learning
GCS: Glasgow coma scale
GBT: Gradient boosted trees
ICU: Intensive care unit
IQR: Interquartile ranges
LR: Logistic regression
MIMIC: Medical information mart for intensive care
SOFA: Sequential organ failure assessment
TRIPOD: Transparent reporting of a multivariable prediction model for individual prognosis or diagnosis
