# Supplementary material for: Early prediction of mortality upon intensive care unit admission
Source: BMC Med Inform Decis Mak. 2024 Dec 18;24:394. doi: 10.1186/s12911-024-02807-6 (PMC11656927; doi:10.1186/s12911-024-02807-6)
Supplement: Supplementary file 1 — Supplementary Material 1 [file 12911_2024_2807_MOESM1_ESM.docx]

**Additional file 1**

Supplement to:

**Early prediction of mortality upon intensive care unit admission**

Yu-Chang Yeh, Yu-Ting Kuo, Kuang-Cheng Kuo, Yi-Wei Cheng, Ding-Shan Liu, Feipei Lai, Lu-Cheng Kuo, Tai-Ju Lee, Wing-Sum Chan, Ching-Tang Chiu, Ming-Tao Tsai, Anne Chao, Nai-Kuan Chou, Chong-Jen Yu, Shih-Chi Ku

Corresponding authors (equal contribution):

Dr. **Yu-Chang Yeh**

Department of Anaesthesiology, National Taiwan University Hospital

No 7, Chung Shan South Road, Taipei, Taiwan

ORCID: 0000-0001-5143-5520

E-mail: [tonyyeh@ntuh.gov.tw](mailto:tonyyeh@ntuh.gov.tw)

Dr. **Shih-Chi Ku**

Department of Internal Medicine, National Taiwan University Hospital

No 7, Chung Shan South Road, Taipei, Taiwan

E-mail: scku1015@gmail.com

**Content**

**1. Information**

**2. Supplementary Tables**

Table S1 Characteristics of patients in the ADM Training and Testing data sets

Table S2 Number of participants with missing data in the ADM data sets

Table S3 Characteristics of patients in the 24H training and testing data sets

Table S4 Characteristics of patients from the MIMIC−IV and CORE database in the 24H sets

Table S5 Number of patients with missing data in the 24H training and testing data sets

Table S6 Performance of models trained with ADM different training datasets on different ADM testing datasets

Table S7 Performance of models trained with 24H different training datasets on different 24H testing datasets

**3. Supplementary Figures**

Figure S1 Performance of ADM and 24H models in the training datasets

Figure S2 Calibration plots of the 24H models

Figure S3 Feature importance of logistic regression models

Figure S4 Feature importance of deep learning models

Figure S5 AUROC of the ADM and 24H models on different days after ICU admission

Figure S6 Personalized risk assessment and simulator

**1. Information**

- **MIMIC IV Code information from https://github.com/MIT-LCP/mimic-iv**

select

ce.subject_id

, ce.stay_id

, ce.charttime

, AVG(case when itemid in (220045) and valuenum > 0 and valuenum < 300 then valuenum else null end) as heart_rate

, AVG(case when itemid in (220179,220050) and valuenum > 0 and valuenum < 400 then valuenum else null end) as sbp

, AVG(case when itemid in (220180,220051) and valuenum > 0 and valuenum < 300 then valuenum else null end) as dbp

, AVG(case when itemid in (220052,220181,225312) and valuenum > 0 and valuenum < 300 then valuenum else null end) as mbp

, AVG(case when itemid = 220179 and valuenum > 0 and valuenum < 400 then valuenum else null end) as sbp_ni

, AVG(case when itemid = 220180 and valuenum > 0 and valuenum < 300 then valuenum else null end) as dbp_ni

, AVG(case when itemid = 220181 and valuenum > 0 and valuenum < 300 then valuenum else null end) as mbp_ni

, AVG(case when itemid in (220210,224690) and valuenum > 0 and valuenum < 70 then valuenum else null end) as resp_rate

, ROUND(

AVG(case when itemid in (223761) and valuenum > 70 and valuenum < 120 then (valuenum-32)/1.8 -- converted to degC in valuenum call

when itemid in (223762) and valuenum > 10 and valuenum < 50 then valuenum else null end)

, 2) as temperature

, MAX(CASE WHEN itemid = 224642 THEN value ELSE NULL END) AS temperature_site

, AVG(case when itemid in (220277) and valuenum > 0 and valuenum <= 100 then valuenum else null end) as spo2

, AVG(case when itemid in (225664,220621,226537) and valuenum > 0 then valuenum else null end) as glucose

FROM mimic_icu.chartevents ce

where ce.stay_id IS NOT NULL

and ce.itemid in

(

220045, -- Heart Rate

225309, -- ART BP Systolic

225310, -- ART BP Diastolic

225312, -- ART BP Mean

220050, -- Arterial Blood Pressure systolic

220051, -- Arterial Blood Pressure diastolic

220052, -- Arterial Blood Pressure mean

220179, -- Non Invasive Blood Pressure systolic

220180, -- Non Invasive Blood Pressure diastolic

220181, -- Non Invasive Blood Pressure mean

220210, -- Respiratory Rate

224690, -- Respiratory Rate (Total)

220277, -- SPO2, peripheral

-- GLUCOSE, both lab and fingerstick

225664, -- Glucose finger stick

220621, -- Glucose (serum)

226537, -- Glucose (whole blood)

-- TEMPERATURE

223762, -- "Temperature Celsius"

223761, -- "Temperature Fahrenheit"

224642 -- Temperature Site

-- 226329 -- Blood Temperature CCO (C)

)

group by ce.subject_id, ce.stay_id, ce.charttime

;

SELECT

MAX(subject_id) AS subject_id

, MAX(hadm_id) AS hadm_id

, MAX(charttime) AS charttime

, le.specimen_id

-- convert from itemid into a meaningful column

, MAX(CASE WHEN itemid = 50862 AND valuenum <= 10 THEN valuenum ELSE NULL END) AS albumin

, MAX(CASE WHEN itemid = 50930 AND valuenum <= 10 THEN valuenum ELSE NULL END) AS globulin

, MAX(CASE WHEN itemid = 50976 AND valuenum <= 20 THEN valuenum ELSE NULL END) AS total_protein

, MAX(CASE WHEN itemid = 50868 AND valuenum <= 10000 THEN valuenum ELSE NULL END) AS aniongap

, MAX(CASE WHEN itemid = 50882 AND valuenum <= 10000 THEN valuenum ELSE NULL END) AS bicarbonate

, MAX(CASE WHEN itemid = 51006 AND valuenum <= 300 THEN valuenum ELSE NULL END) AS bun

, MAX(CASE WHEN itemid = 50893 AND valuenum <= 10000 THEN valuenum ELSE NULL END) AS calcium

, MAX(CASE WHEN itemid = 50902 AND valuenum <= 10000 THEN valuenum ELSE NULL END) AS chloride

, MAX(CASE WHEN itemid = 50912 AND valuenum <= 150 THEN valuenum ELSE NULL END) AS creatinine

, MAX(CASE WHEN itemid = 50931 AND valuenum <= 10000 THEN valuenum ELSE NULL END) AS glucose

, MAX(CASE WHEN itemid = 50983 AND valuenum <= 200 THEN valuenum ELSE NULL END) AS sodium

, MAX(CASE WHEN itemid = 50971 AND valuenum <= 30 THEN valuenum ELSE NULL END) AS potassium

FROM mimic_hosp.labevents le

WHERE le.itemid IN

(

-- comment is: LABEL | CATEGORY | FLUID | NUMBER OF ROWS IN LABEVENTS

50862, -- ALBUMIN | CHEMISTRY | BLOOD | 146697

50930, -- Globulin

50976, -- Total protein

50868, -- ANION GAP | CHEMISTRY | BLOOD | 769895

-- 52456, -- Anion gap, point of care test

50882, -- BICARBONATE | CHEMISTRY | BLOOD | 780733

50893, -- Calcium

50912, -- CREATININE | CHEMISTRY | BLOOD | 797476

-- 52502, Creatinine, point of care

50902, -- CHLORIDE | CHEMISTRY | BLOOD | 795568

50931, -- GLUCOSE | CHEMISTRY | BLOOD | 748981

-- 52525, Glucose, point of care

50971, -- POTASSIUM | CHEMISTRY | BLOOD | 845825

-- 52566, -- Potassium, point of care

50983, -- SODIUM | CHEMISTRY | BLOOD | 808489

-- 52579, -- Sodium, point of care

51006 -- UREA NITROGEN | CHEMISTRY | BLOOD | 791925

-- 52603, Urea, point of care

)

AND valuenum IS NOT NULL

-- lab values cannot be 0 and cannot be negative

-- .. except anion gap.

AND (valuenum > 0 OR itemid = 50868)

GROUP BY le.specimen_id

WITH bg AS

(

select

-- specimen_id only ever has 1 measurement for each itemid

-- so, we may simply collapse rows using MAX()

MAX(subject_id) AS subject_id

, MAX(hadm_id) AS hadm_id

, MAX(charttime) AS charttime

-- specimen_id *may* have different storetimes, so this is taking the latest

, MAX(storetime) AS storetime

, le.specimen_id

, MAX(CASE WHEN itemid = 52028 THEN value ELSE NULL END) AS specimen

, MAX(CASE WHEN itemid = 50801 THEN valuenum ELSE NULL END) AS aado2

, MAX(CASE WHEN itemid = 50802 THEN valuenum ELSE NULL END) AS baseexcess

, MAX(CASE WHEN itemid = 50803 THEN valuenum ELSE NULL END) AS bicarbonate

, MAX(CASE WHEN itemid = 50804 THEN valuenum ELSE NULL END) AS totalco2

, MAX(CASE WHEN itemid = 50805 THEN valuenum ELSE NULL END) AS carboxyhemoglobin

, MAX(CASE WHEN itemid = 50806 THEN valuenum ELSE NULL END) AS chloride

, MAX(CASE WHEN itemid = 50808 THEN valuenum ELSE NULL END) AS calcium

, MAX(CASE WHEN itemid = 50809 and valuenum <= 10000 THEN valuenum ELSE NULL END) AS glucose

, MAX(CASE WHEN itemid = 50810 and valuenum <= 100 THEN valuenum ELSE NULL END) AS hematocrit

, MAX(CASE WHEN itemid = 50811 THEN valuenum ELSE NULL END) AS hemoglobin

, MAX(CASE WHEN itemid = 50813 and valuenum <= 10000 THEN valuenum ELSE NULL END) AS lactate

, MAX(CASE WHEN itemid = 50814 THEN valuenum ELSE NULL END) AS methemoglobin

, MAX(CASE WHEN itemid = 50815 THEN valuenum ELSE NULL END) AS o2flow

-- fix a common unit conversion error for fio2

-- atmospheric o2 is 20.89%, so any value <= 20 is unphysiologic

-- usually this is a misplaced O2 flow measurement

, MAX(CASE WHEN itemid = 50816 THEN

CASE

WHEN valuenum > 20 AND valuenum <= 100 THEN valuenum

WHEN valuenum > 0.2 AND valuenum <= 1.0 THEN valuenum*100.0

ELSE NULL END

ELSE NULL END) AS fio2

, MAX(CASE WHEN itemid = 50817 AND valuenum <= 100 THEN valuenum ELSE NULL END) AS so2

, MAX(CASE WHEN itemid = 50818 THEN valuenum ELSE NULL END) AS pco2

, MAX(CASE WHEN itemid = 50819 THEN valuenum ELSE NULL END) AS peep

, MAX(CASE WHEN itemid = 50820 THEN valuenum ELSE NULL END) AS ph

, MAX(CASE WHEN itemid = 50821 THEN valuenum ELSE NULL END) AS po2

, MAX(CASE WHEN itemid = 50822 THEN valuenum ELSE NULL END) AS potassium

, MAX(CASE WHEN itemid = 50823 THEN valuenum ELSE NULL END) AS requiredo2

, MAX(CASE WHEN itemid = 50824 THEN valuenum ELSE NULL END) AS sodium

, MAX(CASE WHEN itemid = 50825 THEN valuenum ELSE NULL END) AS temperature

, MAX(CASE WHEN itemid = 50807 THEN value ELSE NULL END) AS comments

FROM mimic_hosp.labevents le

where le.ITEMID in

-- blood gases

(

52028 -- specimen

, 50801 -- aado2

, 50802 -- base excess

, 50803 -- bicarb

, 50804 -- calc tot co2

, 50805 -- carboxyhgb

, 50806 -- chloride

-- , 52390 -- chloride, WB CL-

, 50807 -- comments

, 50808 -- free calcium

, 50809 -- glucose

, 50810 -- hct

, 50811 -- hgb

, 50813 -- lactate

, 50814 -- methemoglobin

, 50815 -- o2 flow

, 50816 -- fio2

, 50817 -- o2 sat

, 50818 -- pco2

, 50819 -- peep

, 50820 -- pH

, 50821 -- pO2

, 50822 -- potassium

-- , 52408 -- potassium, WB K+

, 50823 -- required O2

, 50824 -- sodium

-- , 52411 -- sodium, WB NA +

, 50825 -- temperature

)

GROUP BY le.specimen_id

)

, stg_spo2 as

(

select subject_id, charttime

-- avg here is just used to group SpO2 by charttime

, AVG(valuenum) as SpO2

FROM mimic_icu.chartevents

where ITEMID = 220277 -- O2 saturation pulseoxymetry

and valuenum > 0 and valuenum <= 100

group by subject_id, charttime

)

, stg_fio2 as

(

select subject_id, charttime

-- pre-process the FiO2s to ensure they are between 21-100%

, max(

case

when valuenum > 0.2 and valuenum <= 1

then valuenum * 100

-- improperly input data - looks like O2 flow in litres

when valuenum > 1 and valuenum < 20

then null

when valuenum >= 20 and valuenum <= 100

then valuenum

else null end

) as fio2_chartevents

FROM mimic_icu.chartevents

where ITEMID = 223835 -- Inspired O2 Fraction (FiO2)

and valuenum > 0 and valuenum <= 100

group by subject_id, charttime

)

, stg2 as

(

select bg.*

, ROW_NUMBER() OVER (partition by bg.subject_id, bg.charttime order by s1.charttime DESC) as lastRowSpO2

, s1.spo2

from bg

left join stg_spo2 s1

-- same hospitalization

on bg.subject_id = s1.subject_id

-- spo2 occurred at most 2 hours before this blood gas

and s1.charttime between DATETIME_SUB(bg.charttime, INTERVAL 2 HOUR) and bg.charttime

where bg.po2 is not null

)

, stg3 as

(

select bg.*

, ROW_NUMBER() OVER (partition by bg.subject_id, bg.charttime order by s2.charttime DESC) as lastRowFiO2

, s2.fio2_chartevents

-- create our specimen prediction

, 1/(1+exp(-(-0.02544

+ 0.04598 * po2

+ coalesce(-0.15356 * spo2 , -0.15356 * 97.49420 + 0.13429)

+ coalesce( 0.00621 * fio2_chartevents , 0.00621 * 51.49550 + -0.24958)

+ coalesce( 0.10559 * hemoglobin , 0.10559 * 10.32307 + 0.05954)

+ coalesce( 0.13251 * so2 , 0.13251 * 93.66539 + -0.23172)

+ coalesce(-0.01511 * pco2 , -0.01511 * 42.08866 + -0.01630)

+ coalesce( 0.01480 * fio2 , 0.01480 * 63.97836 + -0.31142)

+ coalesce(-0.00200 * aado2 , -0.00200 * 442.21186 + -0.01328)

+ coalesce(-0.03220 * bicarbonate , -0.03220 * 22.96894 + -0.06535)

+ coalesce( 0.05384 * totalco2 , 0.05384 * 24.72632 + -0.01405)

+ coalesce( 0.08202 * lactate , 0.08202 * 3.06436 + 0.06038)

+ coalesce( 0.10956 * ph , 0.10956 * 7.36233 + -0.00617)

+ coalesce( 0.00848 * o2flow , 0.00848 * 7.59362 + -0.35803)

))) as specimen_prob

from stg2 bg

left join stg_fio2 s2

-- same patient

on bg.subject_id = s2.subject_id

-- fio2 occurred at most 4 hours before this blood gas

and s2.charttime between DATETIME_SUB(bg.charttime, INTERVAL 4 HOUR) and bg.charttime

AND s2.fio2_chartevents > 0

where bg.lastRowSpO2 = 1 -- only the row with the most recent SpO2 (if no SpO2 found lastRowSpO2 = 1)

)

select

stg3.subject_id

, stg3.hadm_id

, stg3.charttime

-- raw data indicating sample type

, specimen

-- prediction of specimen for obs missing the actual specimen

, case

when specimen is not null then specimen

when specimen_prob > 0.75 then 'ART.'

else null end as specimen_pred

, specimen_prob

-- oxygen related parameters

, so2

, po2

, pco2

, fio2_chartevents, fio2

, aado2

-- also calculate AADO2

, case

when po2 is null

OR pco2 is null

THEN NULL

WHEN fio2 IS NOT NULL

-- multiple by 100 because fio2 is in a % but should be a fraction

THEN (fio2/100) * (760 - 47) - (pco2/0.8) - po2

WHEN fio2_chartevents IS NOT NULL

THEN (fio2_chartevents/100) * (760 - 47) - (pco2/0.8) - po2

else null

end as aado2_calc

, case

when PO2 is null

THEN NULL

WHEN fio2 IS NOT NULL

-- multiply by 100 because fio2 is in a % but should be a fraction

then 100 * PO2/fio2

WHEN fio2_chartevents IS NOT NULL

-- multiply by 100 because fio2 is in a % but should be a fraction

then 100 * PO2/fio2_chartevents

else null

end as pao2fio2ratio

-- acid-base parameters

, ph, baseexcess

, bicarbonate, totalco2

-- blood count parameters

, hematocrit

, hemoglobin

, carboxyhemoglobin

, methemoglobin

-- chemistry

, chloride, calcium

, temperature

, potassium, sodium

, lactate

, glucose

-- ventilation stuff that's sometimes input

-- , intubated, tidalvolume, ventilationrate, ventilator

-- , peep, o2flow

-- , requiredo2

from stg3

SELECT

MAX(subject_id) AS subject_id

, MAX(hadm_id) AS hadm_id

, MAX(charttime) AS charttime

, le.specimen_id

-- convert from itemid into a meaningful column

, MAX(CASE WHEN itemid = 51221 THEN valuenum ELSE NULL END) AS hematocrit

, MAX(CASE WHEN itemid = 51222 THEN valuenum ELSE NULL END) AS hemoglobin

, MAX(CASE WHEN itemid = 51248 THEN valuenum ELSE NULL END) AS mch

, MAX(CASE WHEN itemid = 51249 THEN valuenum ELSE NULL END) AS mchc

, MAX(CASE WHEN itemid = 51250 THEN valuenum ELSE NULL END) AS mcv

, MAX(CASE WHEN itemid = 51265 THEN valuenum ELSE NULL END) AS platelet

, MAX(CASE WHEN itemid = 51279 THEN valuenum ELSE NULL END) AS rbc

, MAX(CASE WHEN itemid = 51277 THEN valuenum ELSE NULL END) AS rdw

, MAX(CASE WHEN itemid = 52159 THEN valuenum ELSE NULL END) AS rdwsd

, MAX(CASE WHEN itemid = 51301 THEN valuenum ELSE NULL END) AS wbc

FROM mimic_hosp.labevents le

WHERE le.itemid IN

(

51221, -- hematocrit

51222, -- hemoglobin

51248, -- MCH

51249, -- MCHC

51250, -- MCV

51265, -- platelets

51279, -- RBC

51277, -- RDW

52159, -- RDW SD

51301 -- WBC

)

AND valuenum IS NOT NULL

-- lab values cannot be 0 and cannot be negative

AND valuenum > 0

GROUP BY le.specimen_id

;

SELECT

MAX(subject_id) AS subject_id

, MAX(hadm_id) AS hadm_id

, MAX(charttime) AS charttime

, le.specimen_id

-- convert from itemid into a meaningful column

, MAX(CASE WHEN itemid = 50861 THEN valuenum ELSE NULL END) AS alt

, MAX(CASE WHEN itemid = 50863 THEN valuenum ELSE NULL END) AS alp

, MAX(CASE WHEN itemid = 50878 THEN valuenum ELSE NULL END) AS ast

, MAX(CASE WHEN itemid = 50867 THEN valuenum ELSE NULL END) AS amylase

, MAX(CASE WHEN itemid = 50885 THEN valuenum ELSE NULL END) AS bilirubin_total

, MAX(CASE WHEN itemid = 50883 THEN valuenum ELSE NULL END) AS bilirubin_direct

, MAX(CASE WHEN itemid = 50884 THEN valuenum ELSE NULL END) AS bilirubin_indirect

, MAX(CASE WHEN itemid = 50910 THEN valuenum ELSE NULL END) AS ck_cpk

, MAX(CASE WHEN itemid = 50911 THEN valuenum ELSE NULL END) AS ck_mb

, MAX(CASE WHEN itemid = 50927 THEN valuenum ELSE NULL END) AS ggt

, MAX(CASE WHEN itemid = 50954 THEN valuenum ELSE NULL END) AS ld_ldh

FROM mimic_hosp.labevents le

WHERE le.itemid IN

(

50861, -- Alanine transaminase (ALT)

50863, -- Alkaline phosphatase (ALP)

50878, -- Aspartate transaminase (AST)

50867, -- Amylase

50885, -- total bili

50884, -- indirect bili

50883, -- direct bili

50910, -- ck_cpk

50911, -- CK-MB

50927, -- Gamma Glutamyltransferase (GGT)

50954 -- ld_ldh

)

AND valuenum IS NOT NULL

-- lab values cannot be 0 and cannot be negative

AND valuenum > 0

GROUP BY le.specimen_id

SELECT

MAX(subject_id) AS subject_id

, MAX(hadm_id) AS hadm_id

, MAX(charttime) AS charttime

, le.specimen_id

-- convert from itemid into a meaningful column

, MAX(CASE WHEN itemid = 50889 THEN valuenum ELSE NULL END) AS crp

-- , CAST(NULL AS NUMERIC) AS il6

-- , CAST(NULL AS NUMERIC) AS procalcitonin

FROM mimic_hosp.labevents le

WHERE le.itemid IN

(

50889 -- crp

-- 51652 -- high sensitivity CRP

)

AND valuenum IS NOT NULL

-- lab values cannot be 0 and cannot be negative

AND valuenum > 0

GROUP BY le.specimen_id

- **CORE rule of the upper and lower limit of variable**

| **Name** | **Limit** |
| --- | --- |
| Age (years) | 18 - 120 |
| Height (cm) | 100-200 |
| Weight (kg) | 20 - 180 |
| BMI (kg/m²) | 10 - 80 |
| Temperature (°C) | 20 - 46 |
| Heart Rate (beats per minute) | 1 - 300 |
| Respiratory Rate (breaths per minute) | 1 - 80 |
| Systolic Blood Pressure (SBP) (mmHg) | 1 - 350 |
| Diastolic Blood Pressure (DBP) (mmHg) | 1 - 250 |
| Mean Arterial Pressure (MAP) (mmHg) | 1 - 300 |
| Hemoglobin (g/dL) | 1 - 25 |
| White Cell Count (cells/μL) | 0 - 300 |
| Platelet Count (×10³/μL) | 0 - 1500 |
| Sodium Concentration (mmol/L) | 100 - 215 |
| Potassium Concentration (mmol/L) | 0.05 - 15 |
| Creatinine (mg/dL) | 0.1 - 28.3 |
| Blood Urea Nitrogen (mg/dL) | 1.4 - 280 |
| Albumin (g/dL) | 0.5 - 6.5 |
| BIlirubin ( mg/dL ) | 0.05 - 70.2 |
| Blood Sugar (mg/dL) | 0 - 1622 |
| pH | 6.8-7.8 |
| Partial Pressure of Oxygen (PaO₂) (mmHg) | 15-550 |
| Partial Pressure of Carbon Dioxide (PaCO₂) (mmHg) | 10-150 |
| Lactate (mmol/L) | 0-50 |

- **RapidMiner histogram view examples**


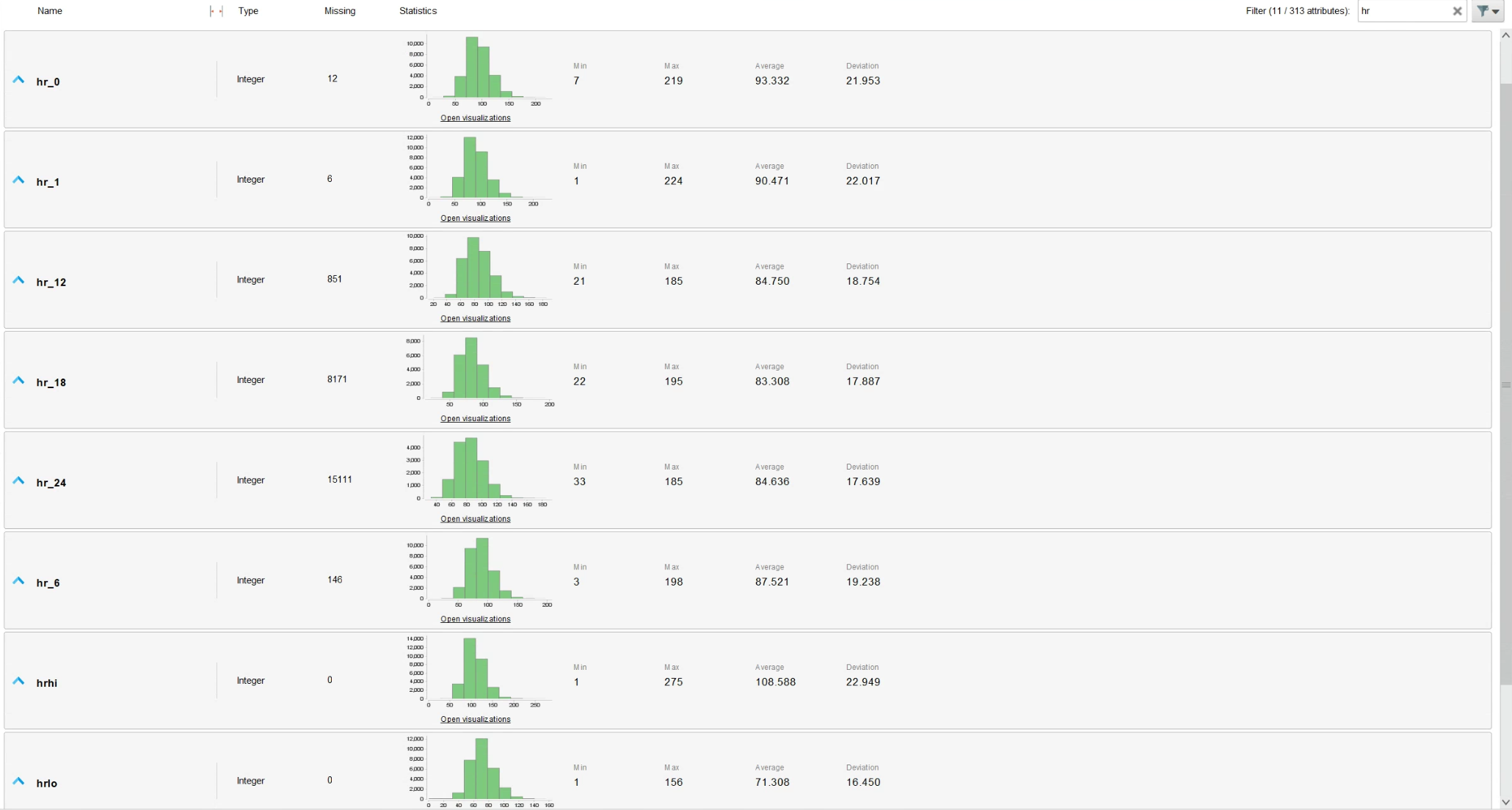


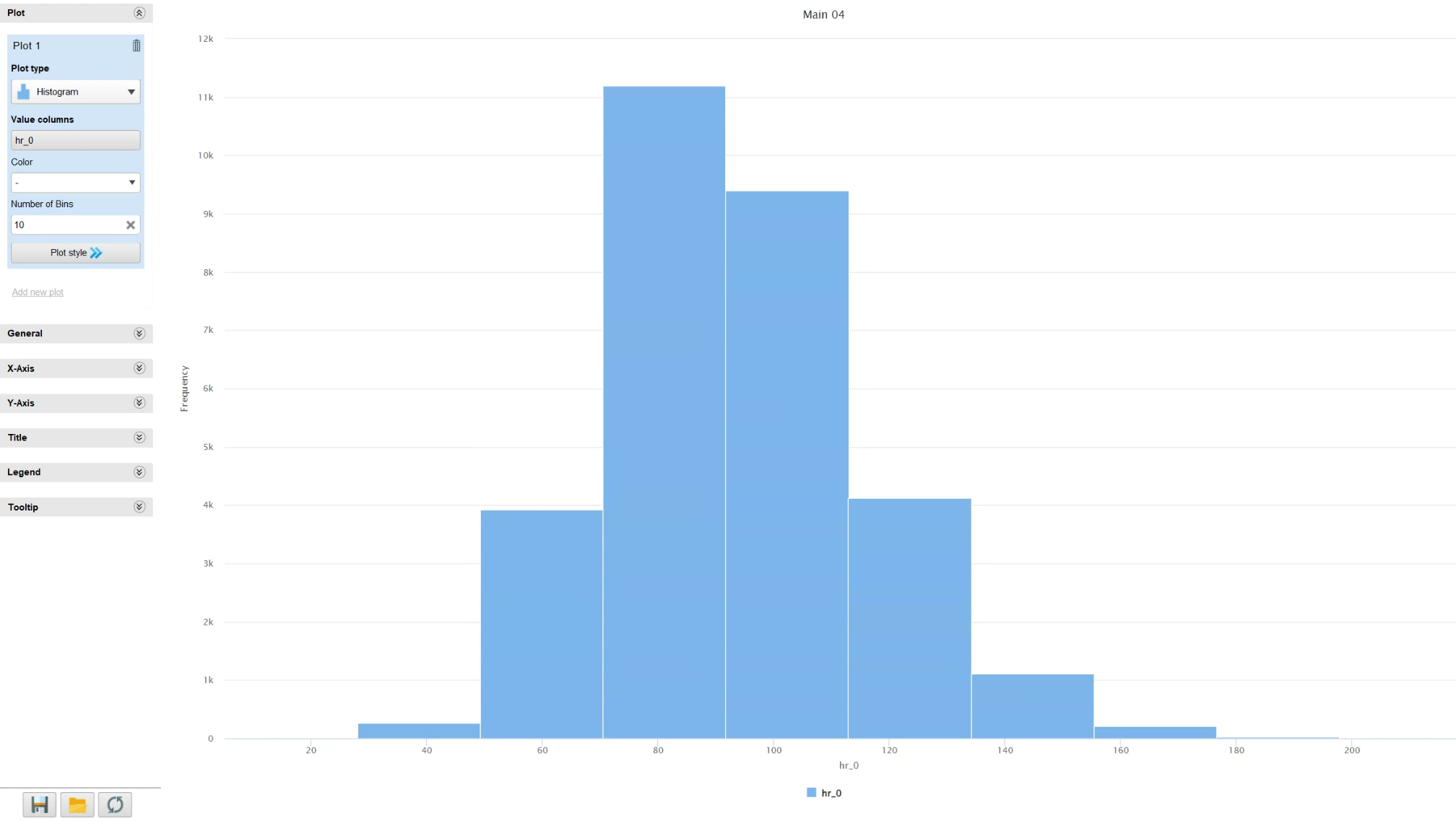


**2. Supplementary tables**

**Table S1 Characteristics of patients in ADM Training and Testing data sets**

|  | **Training** | | | **Testing** | | |
| --- | --- | --- | --- | --- | --- | --- |
|  | Survivors | Nonsurvivors | *p* values | Survivors | Nonsurvivors | *p* values |
| Number | 60925 | 4325 |  | 13395 | 1012 |  |
| Age (years) | 66 (54-77) | 72 (61-82) | <0.001 | 66 (54-76) | 70 (59-81) | <0.001 |
| Female (%) | 26872 (44.1%) | 1944 (44.9%) | 0.288 | 5567 (41.6%) | 404 (39.9%) | 0.307 |
| Height (cm) | 168 (160-175) | 166 (158-175) | <0.001 | 168 (160-176) | 168 (160-175) | 0.139 |
| Weight (kg) | 75 (62.9-90.2) | 71.4 (59.7-86.5) | <0.001 | 75.5 (62.9-90) | 74 (61.7-90) | 0.273 |
| CAD | 9531 (15.6%) | 862 (19.9%) | <0.001 | 2293 (17.1%) | 231 (22.8%) | <0.001 |
| CHF | 15228 (25%) | 1379 (31.9%) | <0.001 | 2740 (20.5%) | 286 (28.3%) | <0.001 |
| ESRD | 12087 (19.8%) | 1090 (25.2%) | <0.001 | 1957 (14.6%) | 207 (20.5%) | <0.001 |
| Diabetes | 18461 (30.3%) | 1363 (31.5%) | 0.097 | 3638 (27.2%) | 282 (27.9%) | 0.626 |
| Dementia | 1955 (3.2%) | 167 (3.9%) | 0.021 | 670 (5%) | 61 (6%) | 0.152 |
| Metastatic cancer | 3832 (6.3%) | 552 (12.8%) | <0.001 | 755 (5.6%) | 82 (8.1%) | 0.001 |
| Postoperative care | 11621 (19.1%) | 249 (5.8%) | <0.001 | 3107 (23.2%) | 73 (7.2%) | <0.001 |
| Elective operation | 6719 (11%) | 53 (1.2%) | <0.001 | 1840 (13.7%) | 12 (1.3%) | <0.001 |
| Pre-ICU hospital stay | 0 (0-1) | 0 (0-2) | <0.001 | 0 (0-1) | 0 (0-2) | <0.001 |
| **Upon ICU admission** | |  |  |  |  |  |
| Glasgow Coma Scale | 15 (12-15) | 10 (4-15) | <0.001 | 15 (9-15) | 8 (3-15) | <0.001 |
| Body temperature (℃) | 36.7 (36.3-37) | 36.6 (36.1-36.9) | <0.001 | 36.7 (36.4-37) | 36.7 (36.3-37) | <0.001 |
| Respiratory rate (bpm) | 18 (15-22) | 21 (17-26) | <0.001 | 18 (15-22) | 21 (18-26) | <0.001 |
| Heart rate (bpm) | 87 (75-101) | 96 (81-114) | <0.001 | 85 (74-99) | 96 (80-113) | <0.001 |
| SBP (mm Hg) | 124 (110-143) | 119 (101-137) | <0.001 | 125 (111-142) | 118 (101-133) | <0.001 |
| DBP (mm Hg) | 69 (58-80) | 66 (54-78) | <0.001 | 69 (60-82) | 68 (56-78) | <0.001 |
| MAP (mm Hg) | 88 (77-100) | 84 (71-98) | <0.001 | 89 (78-101) | 85 (71-96) | <0.001 |
| SOFA score | 4 (2-6) | 10 (6-13) | <0.001 | 4 (2-6) | 11 (7-14) | <0.001 |
| APS | 41 (31-55) | 85 (64-108) | <0.001 | 38 (28-53) | 85 (62-109) | <0.001 |
| LOS in ICU (days) | 2 (1-3) | 3 (1-8) | - | 2 (1-4) | 3 (1-8) | - |
| LOS in hospital (days) | 7 (4-13) | 4 (2-11) | - | 8 (4-15) | 5 (2-12) | - |

Values are presented as numbers (%) or medians (interquartile ranges). *APS* acute physiology score of the acute physiologic assessment and chronic health evaluation (APACHE) III, *bpm* beats or breaths per minute for heart rate and respiratory rate, respectively, *CAD* coronary artery diseases, *CHF* congestive heart failure, *DBP* diastolic blood pressure, *MAP* mean arterial pressure, *ESRD* end-stage renal disease, *ICU* intensive care unit, *LOS* length of stay, *SBP* systolic blood pressure, *SOFA* sequential organ failure assessment.

**Table S2** **Number of participants with missing data in the ADM data sets**

|  | **Training** | | **Testing** | |
| --- | --- | --- | --- | --- |
|  | MIMIC−IV | CORE | MIMIC−IV | CORE |
| Number | 56,478 | 8,772 | 12,103 | 2,304 |
| Height | 30,200 (53.5%) | 844 (9.6%) | 5,721 (47.3%) | 218 (9.5%) |
| Weight | 1,307 (2.3%) | 502 (5.7%) | 260 (2.1%) | 135 (5.9%) |
| Glasgow Coma Scale | 1,870 (3.3%) | 2,857 (21.2%) | 420 (3.5%) | 522 (22.7%) |
| Body temperature | 2,900 (5.1%) | 0 (0%) | 603 (5.0%) | 0 (0%) |
| Respiratory rate | 927 (1.6%) | 14 (0.2%) | 338 (2.8%) | 2 (0.1%) |
| Heart rate | 838 (1.5%) | 1 (0%) | 252 (2.1%) | 3 (0.1%) |
| SBP | 1,737 (3.1%) | 76 (0.9%) | 577 (4.8%) | 18 (0.8%) |
| DBP | 1,737 (3.1%) | 76 (0.9%) | 575 (4.8%) | 18 (0.8%) |
| MAP | 1,626 (2.9%) | 76 (0.9%) | 493 (4.1%) | 18 (0.8%) |

Values are presented as numbers (%). *CORE Center of Outcome and Resource Evaluation critical care database*, *DBP* diastolic blood pressure, *ESRD* end-stage renal disease, *ICU* intensive care unit, *LOS* length of stay, *MAP* mean arterial pressure, *MIMIC* Medical Information Mart for Intensive Care, *SBP* systolic blood pressure, *SOFA* sequential organ failure assessment.

**Table S3 Characteristics of patients in the 24H training and testing data sets**

|  | **Training** | | | **Testing** | | |
| --- | --- | --- | --- | --- | --- | --- |
|  | Survivors | Nonsurvivors | *p* values | Survivors | Nonsurvivors | *p* values |
| Number | 34,383 | 2,912 |  | 8,171 | 704 |  |
| Age (years) | 67 (55-78) | 71 (61-81) | <0.001 | 66 (55-77) | 69 (58-79) | <0.001 |
| Female (%) | 15,052 (43.8%) | 1,276 (43.8%) | 0.966 | 3,413 (41.8%) | 281 (39.9%) | 0.338 |
| Height (cm) | 168 (160-175) | 167 (159-175) | <0.001 | 168 (160-175) | 168 (160-175) | 0.465 |
| Weight (kg) | 75 (62.9-90.2) | 71.1 (59.1-86.5) | <0.001 | 75 (62.8-90) | 74.8 (61.9-91.9) | 0.567 |
| CAD | 5,804 (16.9%) | 589 (20.2%) | <0.001 | 1,427 (17.5%) | 167 (23.7%) | <0.001 |
| CHF | 9,673 (28.1%) | 990 (34%) | <0.001 | 1,837 (22.5%) | 202 (28.7%) | <0.001 |
| ESRD | 7,350 (21.4%) | 763 (26.2%) | <0.001 | 1,279 (15.7%) | 143 (20.3%) | 0.001 |
| Diabetes | 10,975 (31.9%) | 944 (32.4%) | 0.580 | 2,271 (27.8%) | 204 (29%) | 0.502 |
| Dementia | 1,179 (3.4%) | 108 (3.7%) | 0.427 | 427 (5.2%) | 40 (5.7%) | 0.603 |
| Metastatic cancer | 2,176 (6.3%) | 376 (12.9%) | <0.001 | 492 (6%) | 56 (8%) | 0.041 |
| Postoperative care | 6,322 (18.4%) | 178 (6.1%) | <0.001 | 1,625 (19.9%) | 56 (8%) | <0.001 |
| Elective operation | 3,428 (10%) | 44 (1.5%) | <0.001 | 919 (11.2%) | 12 (1.7%) | <0.001 |
| Emergency departments | 11,964 (34.8%) | 1,129 (38.8%) | <0.001 | 1,060 (13%) | 107 (15.2%) | 0.094 |
| Intubated on day 0 or 1 | 4,556 (13.3%) | 766 (26.3%) | <0.001 | 1,143 (14%) | 223 (31.7%) | <0.001 |
| **Upon ICU admission** |  |  |  |  |  |  |
| GCS | 15 (9-15) | 12 (6-15) | <0.001 | 15 (9-15) | 9 (4-15) | <0.001 |
| BT (℃) | 36.7 (36.4-37.1) | 36.6 (36.2-37) | <0.001 | 36.7 (36.4-37) | 37.1 (36.7-37.1) | 0.001 |
| RR (bpm) | 19 (15-22) | 21 (17-26) | <0.001 | 19 (16-22) | 21 (17-26) | <0.001 |
| Heart rate (bpm) | 89 (76-103) | 96 (81-113) | <0.001 | 87 (75-101) | 95 (79-113) | <0.001 |
| SBP (mm Hg) | 125 (109-143) | 121 (103-138) | <0.001 | 125 (110-143) | 122 (105-136) | <0.001 |
| DBP (mm Hg) | 68 (57-79) | 67 (55-79) | 0.002 | 69 (60-82) | 69 (58-80) | 0.053 |
| MAP (mm Hg) | 88 (76-100) | 85 (73-98) | <0.001 | 89 (79-101) | 87 (75-98) | <0.001 |
| **Within 24h** |  |  |  |  |  |  |
| BT_hi (℃) | 37.2 (36.9-37.8) | 37.2 (36.9-37.9) | 0.002 | 37.2 (37-37.7) | 37,2 (37-37.9) | 0.741 |
| Heart rate_hi (bpm) | 104 (90-119) | 113 (97-130) | <0.001 | 102 (90-117) | 114 (96-129) | <0.001 |
| SBP_hi (mm Hg) | 149 (134-166) | 146 (131-163) | <0.001 | 149 (134-165) | 146 (130-165) | 0.012 |
| MAP_hi (mm Hg) | 103 (92-117) | 102 (91-116) | 0.612 | 105 (95-119) | 106 (93-117) | 0.214 |
| GCS_lo | 14 (11-15) | 7 (3-12) | <0.001 | 14 (12-15) | 6 (3-11) | <0.001 |
| Heart rate_lo (bpm) | 70 (60-81) | 76 (63-89) | <0.001 | 68 (59-79) | 73 (61-89) | <0.001 |
| RR_lo (bpm) | 12 (10-14) | 14 (11-16) | <0.001 | 12 (10-14) | 13 (10-16) | <0.001 |
| SBP_lo (mm Hg) | 91 (81-102) | 84 (74-94) | <0.001 | 92 (83-104) | 86 (75-97) | <0.001 |
| Lactate_hi (mmol/L) | 2.4 (1.6-4) | 3.3 (1.9-6.5) | <0.001 | 2.4 (1.6-3.9) | 3.8 (2.1-7.8) | <0.001 |
| hemoglobin_hi (g/dL) | 11.3 (9.8-12.9) | 10.9 (9.5-12.5) | <0.001 | 11.5 (9.9-13.1) | 10.9 (9.1-12.8) | <0.001 |
| WCC_hi (10^9^/L) | 12.1 (8.8-16.7) | 14.1 (9.7-19.8) | <0.001 | 12.6 (9.2-17.1) | 14.9 (10.1-21.6) | <0.001 |
| BUN_hi (mg/dL) | 21 (15-26) | 33 (21-54) | <0.001 | 19 (13-29) | 32 (20-56) | <0.001 |
| Creatinine_hi (mg/dL) | 1.1 (0.8-1.6) | 1.5 (0.9-2.5) | <0.001 | 1 (0.7-1.4) | 1.5 (0.9-2.6) | <0.001 |
| Platelet_hi (10^9^/L) | 212 (157-281) | 195 (126-278) | <0.001 | 202 (156-263) | 172 (105-245) | <0.001 |
| Glucose_hi (mg/dL) | 169 (135-211) | 185 (136-258) | <0.001 | 175 (140-218) | 189 (142-264) | <0.001 |
| Bilirubin (mg/dL) | 0.8 (0.4-1.3) | 0.9 (0.5-2.2) | <0.001 | 0.7 (0.5-1.2) | 1 (0.6-2.6) | <0.001 |
| Sodium_hi (mEq/L) | 139 (137-142) | 140 (136-143) | 0.003 | 140 (137-143) | 141 (136-145) | <0.001 |
| Potassium_hi (mEq/L) | 4.3 (4-4.8) | 4.5 (4.1-5.1) | <0.001 | 4.4 (4-4.9) | 4.6 (4.1-5.2) | <0.001 |
| pH | 7.35 (7.29-7.41) | 7.32 (7.21-7.4) | <0.001 | 7.35 (7.29-7.41) | 7.3 (7.2-7.39) | <0.001 |
| PaO_2_ (mm Hg) | 92 (70-134) | 79 (61-113) | <0.001 | 87 (64-123) | 77 (58-101) | <0.001 |
| WCC_lo (10^9^/L) | 9.3 (6.8-12.6) | 10.4 (6.9-14.8) | <0.001 | 9.7 (7.2-12.9) | 10.7 (7.4-15.2) | <0.001 |
| Platelet_lo (10^9^/L) | 180 (126-242) | 156 (89-227) | <0.001 | 177 (128-232) | 138 (70-195) | <0.001 |
| Albumin_lo (g/dL) | 3.3 (2.8-3.6) | 3 (2.5-3.3) | <0.001 | 3.3 (2.9-3.7) | 3.1 (2.6-3.4) | <0.001 |
| Glucose_lo (mg/dL) | 113 (95-139) | 119 (92-154) | <0.001 | 116 (97-141) | 119 (92-158) | 0.043 |
| SOFA score | 4 (2-6) | 10 (6-13) | <0.001 | 4 (2-6) | 11 (7-14) | <0.001 |
| APS | 41 (31-55) | 85 (64-108) | <0.001 | 38 (28-53) | 85 (62-109) | <0.001 |
| LOS in ICU (days) | 2 (1-3) | 3 (1-8) | - | 2 (1-4) | 3 (1-8) | - |
| LOS in hospital (days) | 7 (4-13) | 4 (2-11) | - | 8 (4-15) | 5 (2-12) | - |

Values are presented as numbers (%) or medians (interquartile ranges). *APS* acute physiology score of acute physiologic assessment and chronic health evaluation (APACHE) III, *bpm* beats or breaths per minute for heart rate and respiratory rate, respectively, *BT* body temperature, *BUN* blood urea nitrogen, *CAD* coronary artery diseases, *CHF* congestive heart failure, *DBP* diastolic blood pressure, *ESRD* end-stage renal disease, *GCS* Glasgow coma scale, *hi* highest, *ICU* intensive care unit, *lo* lowest, *LOS* length of stay, *MAP* mean arterial pressure, *pH* potential of hydrogen, *RR* respiratory rate, *SBP* systolic blood pressure, *SOFA* sequential organ failure assessment, *WCC* white cell count.

**Table S4 Characteristics of patients from the MIMIC−IV and CORE database in the 24H data sets**

|  | **Training** | | **Testing** | |
| --- | --- | --- | --- | --- |
|  | MIMIC−IV | CORE | MIMIC−IV | CORE |
| Number | 31,260 | 6,035 | 7,333 | 1,542 |
| Age (years) | 67 (55-78) | 67 (57-77) | 67 (55-77) | 67 (56-77) |
| Female (%) | 14,020 (44.8%) | 2,308 (38.2%) | 3,120 (42.5%) | 574 (37.2%) |
| Height (cm) | 170 (163-178) | 162 (156-168) | 170 (163-178) | 163 (156-168) |
| Weight (kg) | 78 (65.3-93.2) | 62.3 (53.5-70) | 79 (66-94) | 61.3 (53.3-71) |
| CAD | 5,468 (17.5%) | 925 (15.3%) | 1,366 (18.2%) | 258 (16.7%) |
| CHF | 10,272 (32.9%) | 391 (6.5%) | 1,933 (26.4%) | 106 (6.9%) |
| ESRD | 7,660 (24.5%) | 453 (7.5%) | 1,308 (17.8%) | 114 (7.4%) |
| Diabetes | 9,976 (31.9%) | 1,943 (32.2%) | 1,975 (26.9%) | 50 (32.2%) |
| Dementia | 1,074 (3.4%) | 213 (2.5%) | 417 (5.7%) | 50 (3.2%) |
| Metastatic cancer | 2,005 (6.4%) | 547 (9.1%) | 498 (6.8%) | 50 (3.2%) |
| Postoperative care | 4,106 (13.1%) | 2,394 (39.7%) | 1,044 (14.2%) | 637 (41.3%) |
| Elective operation | 1,719 (5.5%) | 1,753 (29%) | 428 (5.8) | 503 (32.6%) |
| Emergency departments | 10,572 (33.8%) | 2,521 (41.8%) | 547 (7.5%) | 620 (50.2%) |
| Intubated on day 0 or 1 | 1,980 (6.3%) | 3,342 (55.4%) | 540 (7.4%) | 826 (53.6%) |
| **Upon IC admission** |  |  |  |  |
| GCS | 14 (7-15) | 15 (12-15) | 14 (7-15) | 15 (13-15) |
| BT (℃) | 36.7 (36.4-37.1) | 36.4 (36-36.9) | 36.8 (36.5-37.1) | 36.3 (35.8-36.8) |
| RR (bpm) | 18 (15-23) | 19 (16-23) | 19 (16-23) | 19 (16-23) |
| Heart rate (bpm) | 88 (76-103) | 95 (81-110) | 86 (74-100) | 93 (80-109) |
| SBP (mm Hg) | 123 (107-141) | 132 (114-153) | 124 (108-141) | 134 (116-154) |
| DBP (mm Hg) | 67 (56-79) | 71 (60-83) | 70 (59-82) | 72 (60-84) |
| MAP (mm Hg) | 86 (75-99) | 93 (80-105) | 88 (77-101) | 93 (81-106) |
| **Within 24h** |  |  |  |  |
| BT_hi (℃) | 37.1 (36.7-37.7) | 37.3 (36.7-37.9) | 37 (36.7-37/5) | 37.2 (36.6-37.8) |
| Heart rate_hi (bpm) | 103 (90-119) | 109 (96-125) | 102 (89-116) | 108 (95-124) |
| SBP_hi (mm Hg) | 146 (132-163) | 161 (146-180) | 146 (132-161) | 162 (147-180) |
| MAP_hi (mm Hg) | 101 (91-115) | 111 (101-123) | 104 (93-118) | 112 (102-124) |
| GCS_lo | 14 (10-15) | 14 (9-15) | 14 (11-15) | 14 (11-15) |
| Heart rate_lo (bpm) | 70 (60-81) | 72 (62-84) | 68 (59-79) | 71 (61-82) |
| RR_lo (bpm) | 12 (10-15) | 12 (9-14) | 12 (10-14) | 11 (9-13) |
| SBP_lo (mm Hg) | 89 (80-100) | 95 (85-108) | 91 (82-102) | 96 (84-107) |
| Lactate_hi (mmol/L) | 2.1 (1.4-3.4) | 2.4 (1.5-4.1) | 2.3 (1.5-3.6) | 2.4 (1.5-4.1) |
| hemoglobin_hi (g/dL) | 11.2 (9.8-12.9) | 11.3 (9.8-12.9) | 11.4 (9.8-13.1) | 11.5 (9.9-13.1) |
| WCC_hi (10^9^/L) | 12.4 (8.9-17.1) | 11.9 (8.5-16.1) | 13 (9.3-17.8) | 11.7 (8.6-15.5) |
| BUN_hi (mg/dL) | 22 (15-38) | 22 (15-39) | 19 (13-30) | 22 (15-37) |
| Creatinine_hi (mg/dL) | 1.1 (0.8-1.8) | 1 (0.7-1.7) | 1 (0.8-1.5) | 1 (0.7-1.7) |
| Platelet_hi (10^9^/L) | 215 (158-287) | 192 (137-253) | 204 (154-266) | 188 (141-244) |
| Glucose_hi (mg/dL) | 145 (117-192) | 183 (142-243) | 140 (114-180) | 187 (144-245) |
| Bilirubin (mg/dL) | 0.7 (0.4-1.4) | 0.8 (0.6-1.3) | 0.7 (0.4-1.3) | 0.9 (0.6-1.4) |
| Sodium_hi (mEq/L) | 140 (137-142) | 138 (135-140) | 140 (138-143) | 138 (135-140) |
| Potassium_hi (mEq/L) | 4.4 (4-4.9) | 4 (3.7-4.4) | 4.5 (4.1-5) | 4 (3.7-4.4) |
| pH | 7.34 (7.27-7.4) | 7.40 (7.33-7.46) | 7.33 (7.26-7.39) | 7.39 (7.32-7.45) |
| PaO_2_ (mm Hg) | 85 (66-119) | 116 (85-168) | 79 (59-108) | 108 (83-153) |
| WCC_lo (10^9^/L) | 9.2 (6.6-12.6) | 10.3 (7.4-13.9) | 9.7 (7.2-12.9) | 10.2 (7.6-13.7) |
| Platelet_lo (10^9^/L) | 178 (124-243) | 175 (122-238) | 173 (123-231) | 175 (126-226) |
| Albumin_lo (g/dL) | 3.2 (2.7-3.7) | 3.2 (2.8-3.6) | 3.3 (2.7-3.8) | 3.3 (2.9-3.7) |
| Glucose_lo (mg/dL) | 112 (94-135) | 115 (94-142) | 112 (95-133) | 120 (97-148) |
| SOFA score | 5 (3-8) | 8 (5-11) | 4 (2-8) | 8 (5-11) |
| APS | 47 (35-64) | 62 (46-85) | 41 (29-59) | 62 (46-83) |
| ICU mortality | 2313 (7.4%) | 599 (9.9) | 539 (7.4%) | 165 (10.7%) |
| Hospital Mortality | 3651 (11.7%) | 1181 (19.6%) | 818 (11.2%) | 279 (18.1%) |
| LOS in ICU (days) | 3 (2-5) | 4 (2-9) | 3 (2-6) | 4 (2-8) |
| LOS in hospital (days) | 8 (5-14) | 20 (12-38) | 9 (5-16) | 19 (11-34) |

Values are presented as numbers (%) or medians (interquartile ranges). *APS* acute physiology score of the acute physiologic assessment and chronic health evaluation (APACHE) III, *bpm* beats or breaths per minute for heart rate and respiratory rate, respectively, *BT* body temperature, *BUN* blood urea nitrogen, *CAD* coronary artery diseases, *CHF* congestive heart failure, *CORE* Center of Outcome and Resource Evaluation critical care database, *DBP* diastolic blood pressure, *ESRD* end-stage renal disease, *GCS* Glasgow coma scale, *hi* highest, *ICU* intensive care unit, *lo* lowest, *LOS* length of stay, *MAP* mean arterial pressure, *MIMIC* Medical Information Mart for Intensive Care, *pH* potential of hydrogen, *RR* respiratory rate, *SBP* systolic blood pressure, *SOFA* sequential organ failure assessment, *WCC* white cell count.

**Table S5 Number of patients with missing data in the 24H training and testing data sets**

|  | **Training** | | **Testing** | |
| --- | --- | --- | --- | --- |
|  | MIMIC−IV | CORE | MIMIC−IV | CORE |
| Number | 31,260 | 6,035 | 7,333 | 1,542 |
| Height | 13,890 (44.4%) | 707 (11.7%) | 3,041 (41.5%) | 167 (10.8%) |
| Weight | 675 (2.2%) | 409 (6.8%) | 146 (2%) | 101 (6.5%) |
| **Upon IC admission** |  |  |  |  |
| GCS | 1,125 (3.6%) | 1,181 (19.6%) | 215 (2.9%) | 353 (23%) |
| BT | 1,841 (5.9%) | 0 (%) | 347 (4.7%) | 0 (0%) |
| RR | 620 (2.0%) | 10 (0.2) | 215 (2.9%) | 1 (0.1%) |
| Heart rate | 567 (1.8%) | 1 (0%) | 148 (2%) | 2 (0.1%) |
| SBP | 1,176 (3.8%) | 51 (0.8%) | 372 (5.1%) | 13 (0.8%) |
| DBP | 1,191 (3.8%) | 51 (0.8%) | 369 (5%) | 13 (0.8%) |
| MAP | 1,100 (3.5%) | 51 (0.8%) | 316 (4.3%) | 13 (0.8%) |
| **Within 24h** |  |  |  |  |
| BT_hi | 24,106 (77.1%) | 1,385 (22.9%) | 6,221 (84.8%) | 231 (15%) |
| Heart rate_hi | 70 (0.2%) | 0 (0%) | 0 (0%) | 0 (0%) |
| SBP_hi | 176 (0.6%) | 0 (0%) | 30 (0.4%) | 0 (0%) |
| MAP_hi | 77 (0.2%) | 0 (0%) | 4 (0.1%) | 0 (0%) |
| GCS_lo | 14 (0%) | 2 (0%) | 1 (0%) | 0 (0%) |
| Heart rate_lo | 70 (0.2%) | 0 (0%) | 0 (0%) | 0 (0%) |
| RR_lo | 83 (0.3%) | 0 (0%) | 22 (0.3%) | 0 (0%) |
| SBP_lo | 176 (0.6%) | 0 (0%) | 30 (0.4%) | 0 (0%) |
| Lactate_hi | 14,109 (45.1%) | 893 (0.8%) | 3,464 (47.2%) | 210 (13.6%) |
| hemoglobin_hi | 113 (0.4%) | 158 (2.6%) | 69 (0.9%) | 26 (1.7%) |
| WCC_hi | 109 (0.3%) | 229 (3.8%) | 67 (0.9%) | 39 (2.5%) |
| BUN_hi | 83 (0.3%) | 588 (9.7%) | 67 (0.9%) | 104 (6.7%) |
| Creatinine_hi | 81 (0.3%) | 646 (10.7%) | 67 (0.9%) | 139 (9%) |
| Platelet_hi | 112 (0.4%) | 262 (4.3%) | 67 (0.9%) | 82 (5.3%) |
| Glucose_hi | 192 (0.6%) | 325 (5.4%) | 88 (1.2%) | 76 (4.9%) |
| Bilirubin | 13,969 (44.7%) | 1,397 (23.1%) | 3,029 (41.3%) | 240 (15.6%) |
| Sodium_hi | 105 (0.3%) | 183 (3%) | 76 (1%) | 23 (1.5%) |
| Potassium_hi | 120 (0.4%) | 171 (2.8%) | 78 (1.1%) | 21 (1.4%) |
| pH | 11,077 (35.4%) | 412 (6.8%) | 2,972 (40.5%) | 87 (5.6%) |
| PaO_2_ | 11,075 (35.4%) | 407 (6.7%) | 2,972 (40.5%) | 88 (5.7%) |
| WCC_lo | 109 (0.3%) | 229 (3.8%) | 67 (0.9%) | 39 (2.5%) |
| Platelet_lo | 112 (0.4%) | 262 (4.3%) | 67 (0.9%) | 82 (5.3%) |
| Albumin_lo | 17,932 (57.4%) | 2,436 (40.4%) | 3,973 (54.2%) | 613 (39.8%) |
| Glucose_lo | 192 (0.6%) | 325 (5.4%) | 88 (1.2%) | 76 (4.9%) |

Values are presented as numbers (%). *APS* acute physiology score of acute physiologic assessment and chronic health evaluation (APACHE) III, *BT* body temperature, *BUN* blood urea nitrogen, *CORE* Center of Outcome and Resource Evaluation critical care database, *DBP* diastolic blood pressure, *ESRD* end-stage renal disease, *GCS* Glasgow coma scale, *hi* highest, *ICU* intensive care unit, *lo* lowest, *LOS* length of stay, *MAP* mean arterial pressure, *MIMIC* Medical Information Mart for Intensive Care, *pH* potential of hydrogen, *RR* respiratory rate, *SBP* systolic blood pressure, *SOFA* sequential organ failure assessment, *WCC* white cell count.

**Table S6 Performance of models trained with different ADM training datasets on different ADM testing datasets**

| **AUROC values** | | **Testing** | | |
| --- | --- | --- | --- | --- |
| **Training** | **Model** | **CORE** | **MIMIC IV** | **Hybrid** |
| **CORE** | **LR** | 0.879 | 0.785 | 0.801 |
|  | **GBT** | 0.884 | 0.727 | 0.751 |
|  | **DL** | 0.879 | 0.748 | 0.772 |
| **MIMIC IV** | **LR** | 0.843 | 0.823 | 0.820 |
|  | **GBT** | 0.780 | 0.849 | 0.836 |
|  | **DL** | 0.810 | 0.844 | 0.836 |
| **Hybrid** | **LR** | 0.853 | 0.820 | 0.822 |
|  | **GBT** | 0.854 | 0.857 | 0.856 |
|  | **DL** | 0.854 | 0.843 | 0.844 |

*ADM* admission, *AUROC* area under the receiver operating characteristic curve, *CORE* Center of Outcome and Resource Evaluation critical care database, *DL* deep learning, *GBT* gradient boosting trees, *LR* logistic regression, *MIMIC* Medical Information Mart for Intensive Care database.

**Table S7** **Performance of models trained with different 24H training datasets on different 24H testing datasets**

| **AUROC values** | | **Testing** | | |
| --- | --- | --- | --- | --- |
| **Training** | **Model** | **CORE** | **MIMIC IV** | **Hybrid** |
| **CORE** | **LR** | 0.898 | 0.814 | 0.836 |
|  | **GBT** | 0.897 | 0.832 | 0.848 |
|  | **DL** | 0.890 | 0.790 | 0.814 |
| **MIMIC IV** | **LR** | 0.862 | 0.892 | 0.879 |
|  | **GBT** | 0.827 | 0.912 | 0.893 |
|  | **DL** | 0.821 | 0.886 | 0.866 |
| **Hybrid** | **LR** | 0.881 | 0.890 | 0.886 |
|  | **GBT** | 0.894 | 0.914 | 0.910 |
|  | **DL** | 0.881 | 0.893 | 0.890 |

*AUROC* area under the receiver operating characteristic curve, *CORE* Center of Outcome and Resource Evaluation critical care database, *DL* deep learning, *GBT* gradient boosting trees, *LR* logistic regression, *MIMIC* Medical Information Mart for Intensive Care database.

**3. Supplementary Figures**

**Figure S1 Performance of the ADM and 24H models in the training data sets**


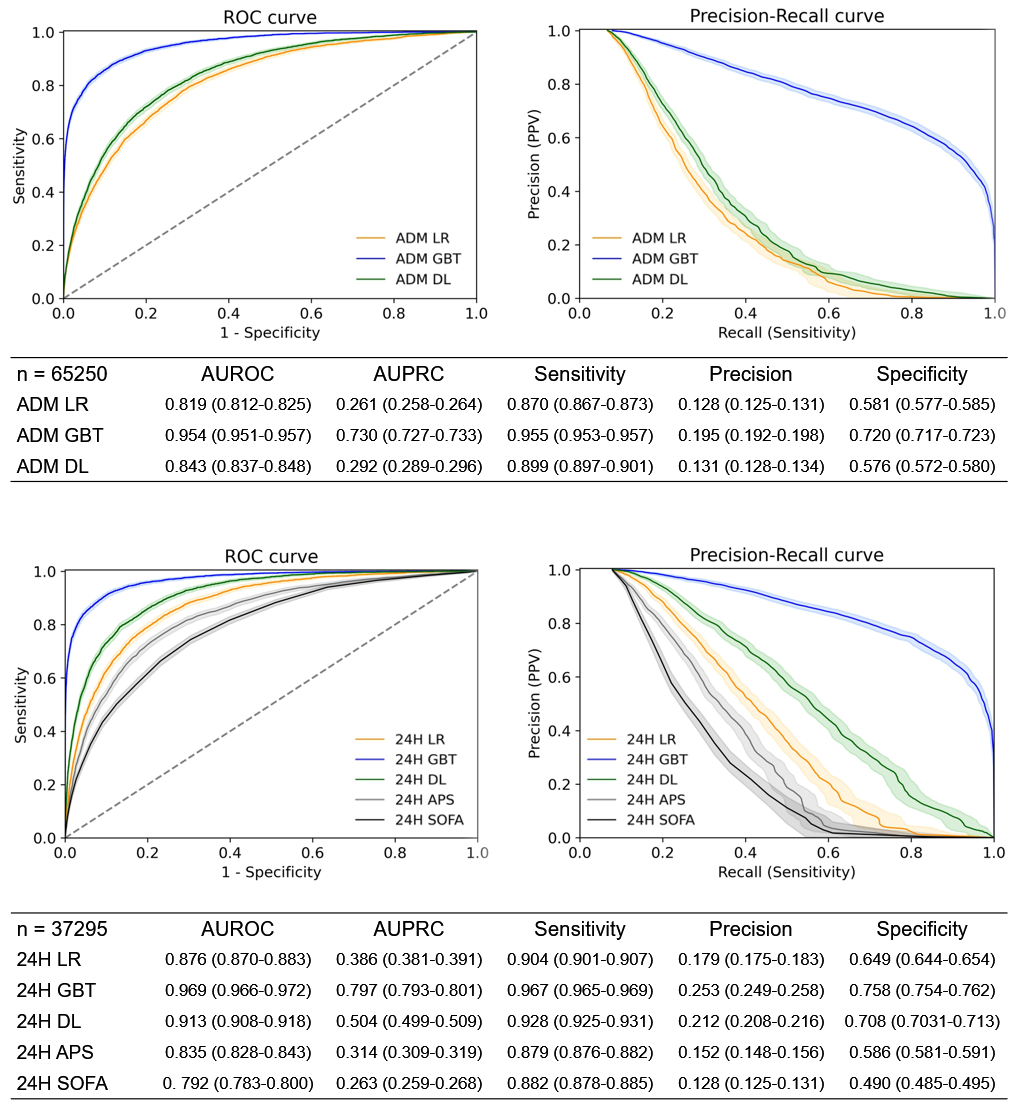


Performance measures are presented as values with corresponding 95% confidence intervals. The cutoff value for sensitivity, precision, and specificity was 0.04 for all models. *ADM* admission, *APS* acute physiology score of the acute physiologic assessment and chronic health evaluation (APACHE) III, *AUPRC* area under the precision-recall curve, *AUROC* area under the receiver operating characteristic curve, *DL* deep learning, *GBT* gradient boosting trees, *LR* logistic regression, *SOFA* sequential organ failure assessment.

**Figure S2 Calibration plots of the 24H models**

**
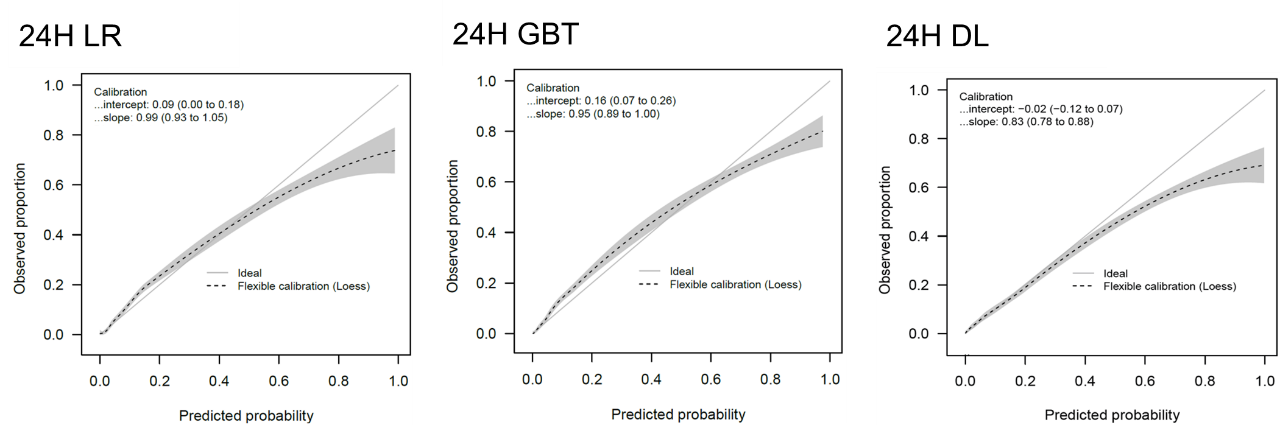
**

*DL deep learning, GBT gradient boosting trees, LR logistic regression.*

**Figure S3 Feature importance of logistic regression models**


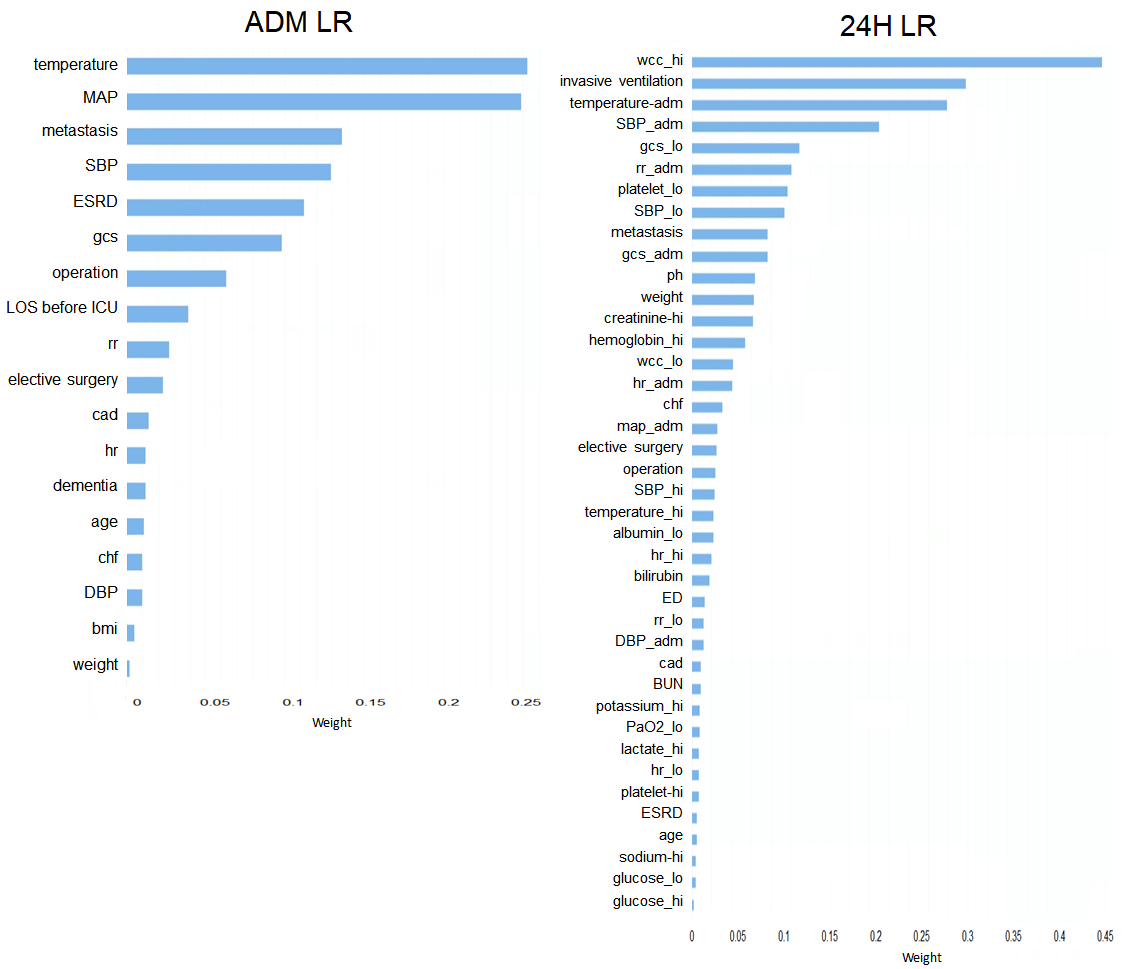


*ADM* admission, *bmi* body mass index, *BUN* blood urine nitrogen, *cad* coronary artery diseases, *chf* congestive heart failure, *DBP* diastolic blood pressure, *ED* emergency department, *ESRD* end stage renal disease, *gcs* Glasgow coma scale, *hr* heart rate, *ICU* intensive care unit, *inv* invasive ventilation, *LOS* length of stay, *LR* logistic regression, *MAP* mean arterial pressure, *ph* power of hydrogen, *plat* platelet, *rr* respiratory rate, *SBP* systolic blood pressure, *wcc* white cell counts.

**Figure S4 Feature importance of deep learning models**


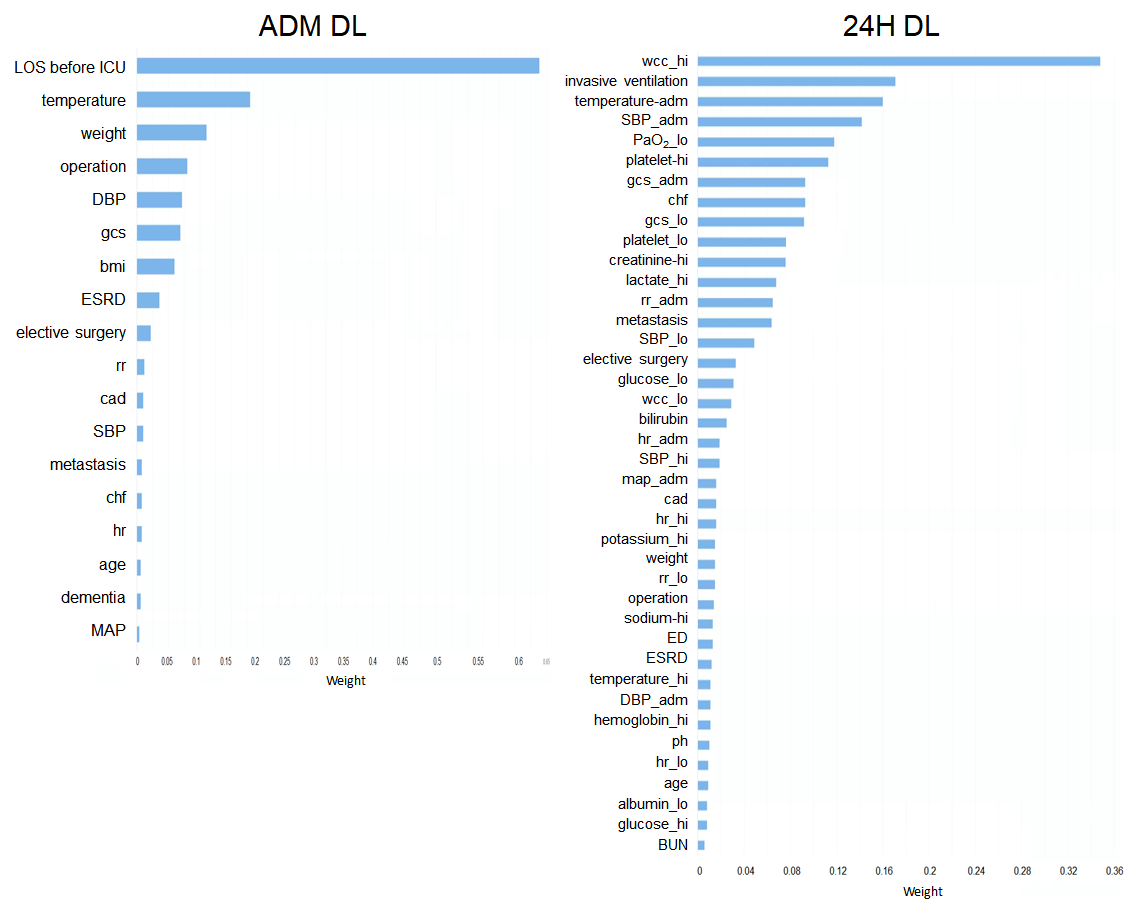


*ADM* admission, *bmi* body mass index, *BUN* blood urine nitrogen, *cad* coronary artery diseases, *chf* congestive heart failure, *DBP* diastolic blood pressure, *DL* deep learning, *ED* emergency department, *ESRD* end stage renal disease, *gcs* Glasgow coma scale, *hr* heart rate, *ICU* intensive care unit, *inv* invasive ventilation, *LOS* length of stay, *MAP* mean arterial pressure, *ph* power of hydrogen, *plat* platelet, *rr* respiratory rate, *SBP* systolic blood pressure, *wcc* white cell counts.

**Figure S5 AUROC of the ADM and 24H models on different days after ICU admission**


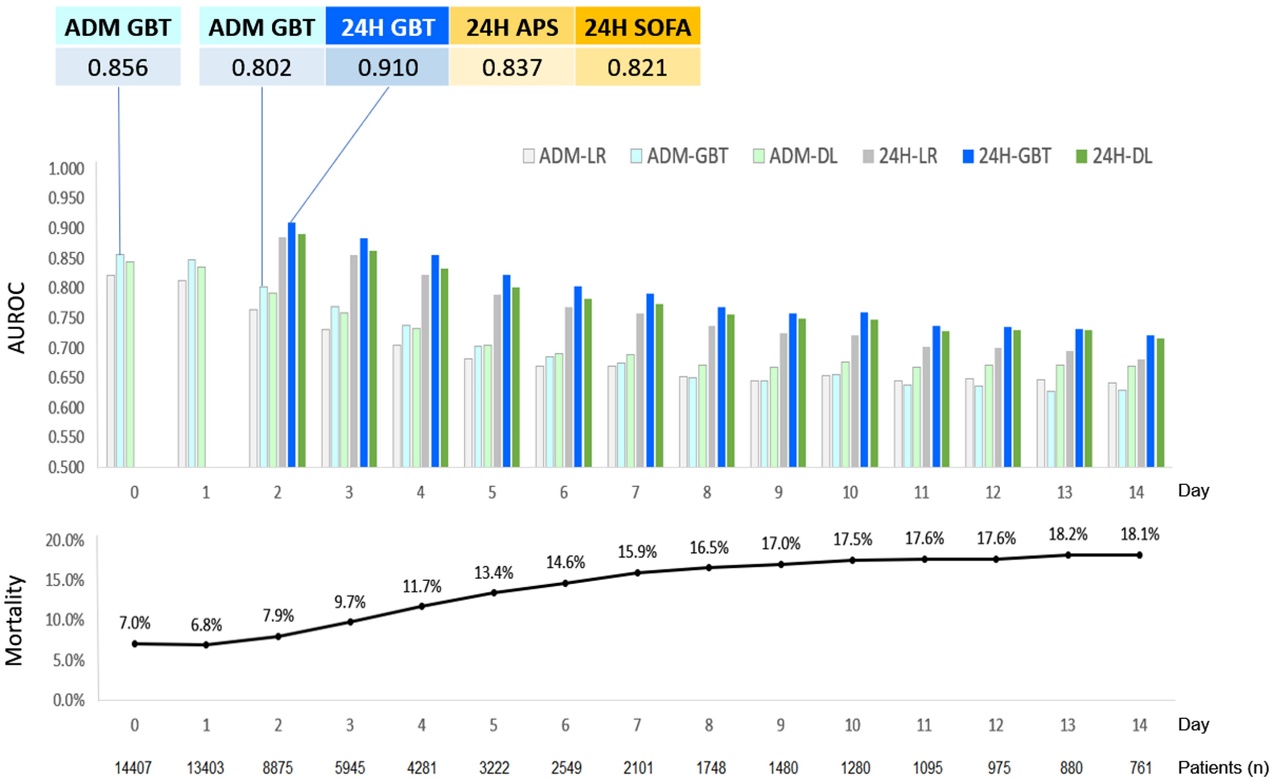


*The AUROC values of the ADM and 24H models and mortality rate of patients who remained in the ICU on day 0 to 14 of ICU admission. ADM admission, APS acute physiology score of the acute physiologic assessment and chronic health evaluation (APACHE) III, AUROC area under the receiver operating characteristic curve, DL deep learning, GBT gradient boosting trees, ICU intensive care unit, LR logistic regression, SOFA sequential organ failure assessment.*

**Figure S6 Personalized risk assessment and simulator**


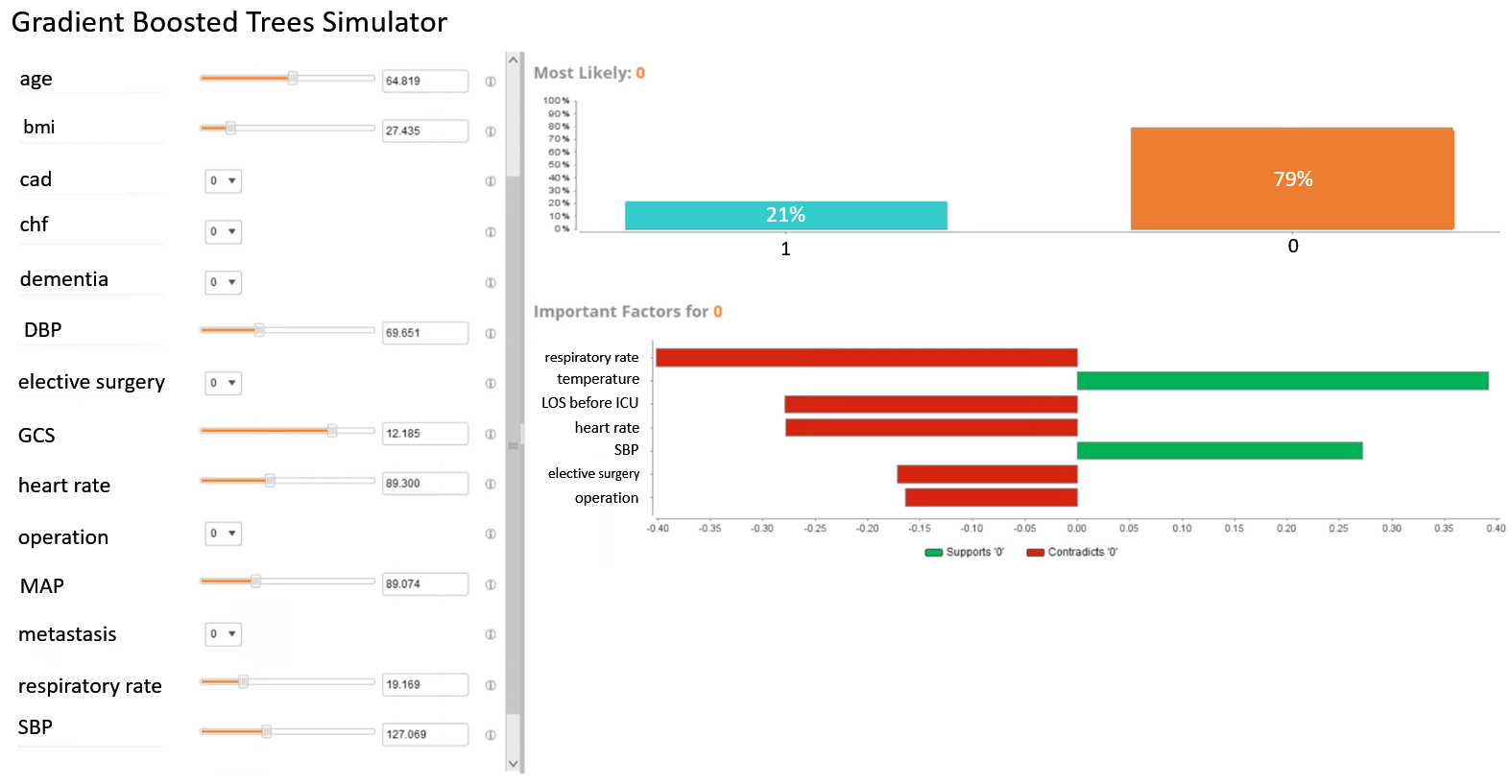


Personal risk is assessed with the contribution of each important factor. If the value of any risk factor was changed in the gradient boosted trees algorithm simulator, the simulator recalculated the predicted risk accordingly. *bmi* body mass index, *cad* coronary artery diseases, *chf* congestive heart failure, *DBP* diastolic blood pressure, *GCS* Glasgow coma scale, *MAP* mean arterial pressure, *SBP* systolic blood pressure.
